# Measuring Individual Differences in Decision Biases: Methodological Considerations

**DOI:** 10.3389/fpsyg.2015.01770

**Published:** 2015-11-19

**Authors:** Balazs Aczel, Bence Bago, Aba Szollosi, Andrei Foldes, Bence Lukacs

**Affiliations:** ^1^Institute of Psychology, Eotvos Lorand UniversityBudapest, Hungary; ^2^Paris Descartes UniversityParis, France; ^3^Corvinus University of BudapestBudapest, Hungary

**Keywords:** decision making, heuristics and biases, individual differences, decision biases, multiple-bias questionnaires

## Abstract

Individual differences in people's susceptibility to heuristics and biases (HB) are often measured by multiple-bias questionnaires consisting of one or a few items for each bias. This research approach relies on the assumptions that (1) different versions of a decision bias task measure are interchangeable as they measure the same cognitive failure; and (2) that some combination of these tasks measures the same underlying construct. Based on these assumptions, in Study 1 we developed two versions of a new decision bias survey for which we modified 13 HB tasks to increase their comparability, construct validity, and the participants' motivation. The analysis of the responses (*N* = 1279) showed weak internal consistency within the surveys and a great level of discrepancy between the extracted patterns of the underlying factors. To explore these inconsistencies, in Study 2 we used three original examples of HB tasks for each of seven biases. We created three decision bias surveys by allocating one version of each HB task to each survey. The participants' responses (*N* = 527) showed a similar pattern as in Study 1, questioning the assumption that the different examples of the HB tasks are interchangeable and that they measure the same underlying construct. These results emphasize the need to understand the domain-specificity of cognitive biases as well as the effect of the wording of the cover story and the response mode on bias susceptibility before employing them in multiple-bias questionnaires.

## Introduction

The heuristics and biases (HB) literature has produced a wide collection of tasks to demonstrate the systematic deviation of people's thinking from rational thought. These bias-assessment tasks are frequently employed in the field of judgment and decision making to study differences between and within groups and individuals. Measuring individual differences in the susceptibility to decision biases has become a targeted research question since it was suggested that there is an unexplored variance in rational thinking independent of cognitive abilities and intelligence (Stanovich and West, 1998, 2001; Stanovich, 1999). This notion bears relevance to the question of whether intelligence tests encompass all important aspects of rational thinking (Stanovich, 2012), or rather rationality deserves an additional assessment tool (Stanovich et al., 2011). A methodology that allows for the exploration of individual differences in cognitive biases can also help us understand how susceptible people are to the individual decision biases; whether there are independent factors behind these biases; and how effective certain debiasing methods are. Experimental settings devised for studying these questions often attempt to include a wide range of these tasks to test them in a within-subject design. The format and structure of the HB tasks, however, vary greatly, as they have been developed independently, and judgment and decision-making researchers tend to select tasks for habitual rather than empirical reasons. Nevertheless, when analyzed together, several methodological issues should be taken into consideration. In this paper, we highlight a list of methodological challenges that should be faced when measuring several HB tasks together with the aim of assessing the degree of susceptibility on an individual level, or when using performance measures of these tasks as an indicator of their shared underlying factors.

### Individualized scores

The assumption that the HB tasks share some underlying cognitive properties prompts researchers to create a composite score from the performance measures of the individual tasks (e.g., Bruine de Bruin et al., 2007; Toplak et al., 2007). Similarly, when studying the association of performance on HB tasks with other psychological factors, within-subject design is required where the performance of the participants is evaluated individually. Unfortunately, when evaluating the traditional HB tasks it is not always straightforward whether the given person violated a normative rule or not in his or her decision. Sometimes the bias can be assessed only in comparison to another decision or on a group level. From this aspect, we find that the HB tasks that measure individual biases fall into three categories.

Type-A: In this type of task, a single question is diagnostic to the person's susceptibility to violating the given normative rule. For example, in the standard tasks of the Conjunction fallacy (Tversky and Kahneman, 1983), or the Base-rate neglect (Bar-Hillel, 1980) certain answers always indicate suboptimal reasoning. For measurement purposes, sometimes the bias is calculated from an amalgamation of several versions of the question (e.g., overconfidence, Lichtenstein and Fischhoff, 1977).

Type-B: For these tasks, the answer to the given question can be regarded as indicating the person's decision bias only in relation to another decision of the individual. Therefore, the task is made of two questions in a within-subjects design. For example, the Framing effect is sometimes measured on the level of the individual by comparing the answers given to two differently framed versions of the same question within the same questionnaire (e.g., Resistance to framing in Toplak et al., 2014).

Type-C: This type of task also assesses the presence of bias by two questions, but on a group level in between-subjects design. For example, the Status Quo bias is typically studied in a way that two groups would receive the same question, but one group would also know that one of the options is the current state of affairs (e.g., Samuelson and Zeckhauser, 1988).

A main challenge for measuring individual differences with HB questions is to reconstruct Type-C tasks into Type-A or Type-B designs. For example, the Hindsight bias is often measured in two groups. In a typical hindsight bias experiment, both groups receive descriptions of a number of events, each with a few possible outcomes and the participants are asked to indicate the likelihood of the given outcomes. For one group, however, the outcomes that actually occurred are indicated. The estimate of this group (the hindsight probability estimate) is compared to the estimate of the foresight group, which was not told about the actual outcomes. Typically, the mean estimate of the hindsight group is higher than the foresight group (Christensen-Szalanski and Willham, 1991), indicating an effect of outcome knowledge in the participants' judgments. Although this experimental design provides a sensible arrangement for the demonstration of the hindsight bias, it is unable to offer a measure for the purpose of individual difference analyses. A noteworthy attempt to provide an individualized score for Hindsight bias can be found in a study conducted by Stanovich and West (1998). In Experiment 4, they asked the participants to read two forms of 35 general knowledge questions. For the first form of questions, the correct answers were not indicated and they were asked to indicate their confidence in their responses. For the second set of questions, the correct answers were indicated and they were asked to indicate the probability that they would have answered the questions correctly. The forms were counter-balanced and the scores on the two forms were standardized based on their distributions. From these measures, the authors created an individualized hindsight score by subtracting for each individual the percentage of their correct responses on the knowledge calibration test from their percentage estimate on the hindsight test. This is a notable attempt to create a Type-B from a Type-C HB task, yet taking a closer look it might not satisfy expectations. The degree of noise on one of the forms does not necessarily correspond with the degree of noise on the other form, as an individual might actually know the answers on the hindsight form and be less confident about the answers on the foresight form. Therefore, this attempt to solve the Type-C problem does not provide the score necessary for individual difference analyses. Indices of group performance are not adequate to serve as reference points for evaluating rationality at the level of the individual.

A more promising technique to solve the Type-C problem is asking the two versions of the question from the same people, instead of testing them in two groups. This solution can be applied to the Hindsight bias by asking the participants to indicate a range of possible values for a question (Hardt and Pohl, 2003) or to give a confidence rating for their answer (Teovanović et al., 2015). Then, in a later phase, they have to recall these estimates immediately after receiving feedback for the initial question. The Framing effect has also been shown to be observable in a within-subject design (Frisch, 1993). Here, researchers prefer to place the two versions of the questions in distant parts of the questionnaire (e.g., Parker and Fischhoff, 2005), or insert a longer delay between them (e.g., 1 week in Levin et al., 2002) to decrease the effect of the memory of the first question on the second one. With higher resemblance between the two versions of the questions, it becomes more difficult to camouflage the link between the two items. In fact, the framing effect is consistently less prevalent in within-subjects comparisons than in between-subjects design (Gambara and Piñon, 2005).

Another difficulty in adapting Type-C tasks to Type-B formats occurs in cases where the effect of the current state of affairs is measured. For example, one typical way to demonstrate the status quo bias is to inform only one of two groups about the current state of affairs regarding the same decision situation. In consecutive presentation of the two questions in a within-subject arrangement, either the neutral or the status quo description must come first, and thus the results can become biased. When the neutral question precedes, the decision on this question can become the status quo; when the status quo question comes first, it can affect the interpretation of the neutral description coming later. Roca et al. (2006) described a different approach to test within-subject status quo effect. They asked the participants to choose between different ambiguous gambles where the proportion of the number of balls of two colors in the urn was unknown and they won if they drew the ball of their chosen color. Before playing the gamble, they were offered an opportunity to exchange those gambles for their non-ambiguous counterparts. This decision was contrasted with a consecutive neutral context where the participants had to choose between ambiguous and unambiguous gambles to play. Within-subject status quo bias was defined by the behavior of retaining the ambiguous gamble in the first context, but choosing the unambiguous gamble in the neutral condition. Along with demonstrating the effect of status quo bias, a strong tendency of the participants was observed to choose consistently between the contexts (65–92% of the decisions), probably due to the consecutive presentation of the two situations.

In summary, the adaption of Type-C questions to Type-B tasks is a persistent challenge for measuring individual differences in HB tasks. An added difficulty is that even when this adaption is successful people are more immune to violating the principle of invariance (Tversky and Kahneman, 1986) and tend to choose consistently (LeBoeuf and Shafir, 2003). In fact, empirical analyses show that the within-subject effect is much weaker than the between-subject effect when the expectation of decision consistency may override their default answer (Gambara and Piñon, 2005; Roca et al., 2006).

### Construct validity

Another requirement for establishing the degree to which individuals are biased in their decisions is that the incorrect answers should be due to the given cognitive bias that the question was devised to measure. In the related literature, we found surprisingly numerous cases where not all of the incorrect questions are good examples of the studied bias.

For example, West et al. (2008) describe their Gambler's Fallacy task and its scoring such as follows (pp. 932–933):

*When playing slot machines, people win something about 1 in every 10 times. Lori, however, has just won on her first three plays. What are her chances of winning the next time she plays? Choose the best answer*.

The problem was followed by the choices: (a) She has better than 1 chance in 10 of winning on her next play, (b) She has < 1 chance in 10 of winning on her next play, (c) She has a 1 chance in 10 that she will win on her next play. The correct response of c was scored as 1, while any other response incorrect and scored as 0.

In this example, response (b) would indicate that the participant followed the pattern predicted by the Gambler's Fallacy: if winning happened more frequently than normal then we should expect less than normal frequency to follow. However, response (a), while being incorrect, is more similar to what the hot-hand illusion (Gilovich et al., 1985) would predict, since a sequence of success is followed by increased likelihood of success. Consequently, 0 score on this question is not a valid indicator of the studied effect. Methodological problems remain present when researchers define which concrete, incorrect response would indicate the presence of the given bias. For example, Toplak et al. (2011) measured the Sunk Cost effect in two parts (p. 1289.):

“In the first part, participants are told to imagine that they are staying in a hotel room, and they have just paid $6.95 to see a movie on pay TV. Then they are told that they are bored 5 min into the movie and that the movie seems pretty bad. They are then asked whether they would continue to watch the movie or switch to another channel. In the second part, the scenario is analogous, except that they have not had to pay for the movie. They are asked again whether they would continue to watch the movie or switch to another channel. Responses were scored as correct if the participant chose consistently across the two situations (either continuing to watch the movie in both cases, or switching to another channel in both cases), and as incorrect if the participant displayed a sunk cost (that is, continuing to watch the movie if it had been paid for but not if it was free).”

In this example, out of the four possible choices of the participant, two were scored as correct, but only one of the two remaining possible choices was scored as incorrect, the one that indicates the effect of Sunk Cost. Nevertheless, the possible case when the participant would continue to watch the movie if it hasn't been paid for, but would not watch it if it has been paid for was not scored as either correct or incorrect. It is unstated how these items were analyzed, but when the aim is to create a composite score from the HB tasks then this kind of scoring becomes problematic. Scoring these responses as 0 or disregarding the item from the composite score would both bias its validity. Discarding the participant's data from the analysis if he or she selects this combination of choices would be impractical for the aim of creating comparable within-subjects measures.

### Comparability

Variation in susceptibility to different biases is a recurring question of the decision-making research program (Blais and Weber, 2001; Toplak et al., 2011). To compare the degree of susceptibility in the different biases, we have to be able to assume that the response mode of the tasks does not bias the sensitivity of the task to detect the corresponding bias, so that the chance of giving correct (and incorrect) responses for the different tasks is the same. This criterion is mostly never satisfied in studies testing HB tasks together. The chance of correct choice can be as high as 50% where the participants can choose from only between two options (e.g., the Base-rate neglect problem in Toplak et al., 2011), but in the case of open questions, this chance can be also infinitely low (e.g., the Gambler's fallacy problem in Toplak et al., 2011). Higher chance for correct choice decreases the sensitivity of the question to measure the given bias and creates a confound when studying the variance in susceptibility among the biases. In addition, a composite score created from tasks with unadjusted sensitivity levels can underestimate the effect of less sensitive HB tasks.

### Motivation

One of the criticisms that the HB research program received over the course of years is that the suboptimal performance on the cognitive bias tasks might be partly due to participants being under-motivated to allocate the necessary cognitive effort to solving the questions (Klein, 1999). In fact, one assumption behind reasoning models is that humans are “cognitive misers” (Simon, 1955; Evans and Stanovich, 2013): they are reluctant to assign effort to a task, unless it is important to them. Financial incentives were found to achieve only limited improvement in performance (Camerer and Hogarth, 1999), while rather the ecological validity of the questions increased interest in the issue. From an ecological validity perspective, HB tasks are different from real life decisions since they are made in laboratories about often artificial questions or hypothetical situations (Gigerenzer et al., 1999; Klein, 1999). While it is hard to link performance to real-life consequences (other than payment) when using questionnaires, the descriptions of the questions rarely indicate that the outcome of the decision would have, even fictional, critical consequences for or relevance to the participant's personal aims.

### Aim of the study

This study represents an attempt to increase the validity and reduce the inconsistency of multiple-bias questionnaires. We aimed to test whether different versions of the same bias task are interchangeable. Within-subject arrangements cannot exclude that one task or the answer to that would not make people answer similarly on other analogous questions, so we tested this assumption in a between-subject design. We created two versions of the same survey and tested each with a random group of people. Next, we analyzed the psychometric properties of the surveys, and we compared the correspondence between the measured susceptibility to the individual biases with their performance on the Cognitive Reflection Test (CRT; Frederick, 2005), as it is a potent predictor of performance on HB tasks (Toplak et al., 2011). By these correlations and by a Factor Analysis we aimed to assess the coherence within and the consistency between the bias surveys.

## Study 1

### Methods

#### Participants

One thousand two hundred and seventy nine participants (697 female) were recruited, comprising mostly university students, native speakers of Hungarian with a mean age of 22.96 years (*SD* = 8.14). Leaders of Hungarian student organizations were asked to circulate a recruiting e-mail on their respective mailing-lists. The e-mail contained a link to the online questionnaire. The participants were motivated to take part in the survey by a 50.000 HUF prize (approx. 180 USD) drawn from among those who completed the survey. Participants, who wanted to take part in the lottery, were identified by their e-mail address, which they could provide voluntarily. The research was approved by the institutional ethics committee of Eotvos Lorand University, Hungary.

One hundred two participants' results were excluded from the analysis as their answers clearly indicated that they did not understand the questions or were not motivated to answer sensibly^1^. Missing data were omitted in a pairwise manner.

#### Materials

Two versions of an HB task battery were administered. Both questionnaires contained the same popularly tested bias-tasks (Anchoring effect, Base-rate neglect, Conjunction fallacy, Covariation detection, Framing effect, Gambler's fallacy, Insensitivity to sample size, Monty Hall problem, Outcome bias, Probability match, Regression to the mean, Relativity bias, Sunk cost fallacy^2^), the only difference between the two versions being the wording of the questions. All testing materials were presented in Hungarian. A pilot test was conducted with volunteers to improve sensitivity and comprehensibility in which they were able to report any issues in the tasks that prevented them from fully understanding the situations. The tasks are available in the Supplementary Materials (Study 1, Extended Methods section and Table 1) for both tests.

**Table 1:** **An example of the adapted changes on the Gambler's Fallacy task**.

**Original task**	**Modified task**
When playing slot machines, people win something 1 out of every 10 times. Julie, however, just won her first three plays. What are her chances of winning the next time she plays? ___ out of ___ [An answer of 1 out of 10 is the normative response and was scored as 1, while any other response was scored as 0.] (Toplak et al., 2007, p. 111)	You are responsible for the financial planning of a real estate broking firm Based on past data, your entrusted broker company makes profitable deals in 60% of the cases in the long term They were unsuccessful with their last nine cases. What are the chances that the 10th case will be successful? (A) 60%; (B) 70%; (C) 80%; (D) 90% [Correct answer: (A)]

As the tasks were adapted from the literature, we found it important to put notable emphasis on the issues outlined above, namely comparability, construct validity and motivation. Thus, firstly, the probability of randomly giving the correct answer was fixed at 25% for each question. Secondly, the answer options were constructed so that they measure only the bias they are supposed to measure. Finally, to increase the participants' motivation to find a good solution to the presented problems, we reframed the traditional HB tasks so that the participants should envisage themselves in the situation of a concrete decision maker where the outcome of the decision would be critical for them. We also decided to place all of the questions in one domain. Among the decision-making domains with probably the most critical outcomes are military, medical, aviation and managerial domains. We speculated that out of these, the situations described in the managerial domain are the most understandable for the widest range of participants. Table 1 shows an example of the adapted changes on the HB tasks, such as the managerial theme, unified level of chance, and the description of critical situations in order to increase motivation.

#### Procedure

Participants were assigned one of the two tests via arbitrary sampling. After obtaining informed consent, they were asked to provide basic demographic data. Next, they completed the survey consisting of the HB tasks in a fixed order and the three items of the original CRT (Frederick, 2005). For each HB question, a limit constrained the participant's time to provide a response so that all participants would spend approximately the same amount of time answering the questions without being able to seek external help. To make sure that participants could properly understand the situation, they were first presented with the description with no time-pressure. After they indicated that they understood the situation and were ready continue, the answer options were revealed along with an indication of how much time they have left until they needed to give an answer. The limit ranged between 30 and 70 s based on the results of pilot testing. At the end of the experiment, participants received personal feedback of their results alongside a brief description of each task.

#### Scoring

For each HB task participants scored either 1 for the correct or 0 for the incorrect answer. Composite score was calculated as the sum of the scores of the HB tasks. CRT tasks were also scored 1 for correct and 0 for incorrect answers. Composite scores for the CRT tasks were calculated the same way as for the HB tasks.

### Results

#### Reliability

Reliability measures showed very low internal consistency for the composite scores on both of the tests (Table 2). Both Cronbach's alpha and Split-half reliability measures were below the acceptable level. This low internal consistency questions whether items measure a single unidimensional latent construct.

**Table 2:** **Reliability measures of the HB composite score**.

	**Split-half**	**Cronbach's alpha**
Test 1	0.38	0.37
Test 2	0.21	0.23

#### Factor analysis

A polynomial Factor Analysis with an oblique (Promax) rotation method, and with a Diagonal Weighted Least Square estimation procedure was conducted on the data, using the psych R package (Revelle, 2014). The two tests were analyzed separately; on the first test the best fitting factor structure was assessed, then an analysis with the same number of factors was conducted on the second test.

As a first step, it was tested whether the data are adequate for Factor Analysis. With regard to Test 1, the Kaiser-Meyer-Olkin factor (KMO; computed on the polychoric correlation matrix) reached an acceptable value, KMO = 0.66, and the Bartlett's test also indicated that the correlation matrix is not an identity matrix, $\chi_{\left(78\right)}^{2}=620.32$, *p* < 0.001. In Test 2, similar results were observed, with regard to the KMO = 0.62, and to the Bartlett's test, $\chi_{\left(78\right)}^{2}=746.34$, *p* < 0.001. These results suggested that the data are suitable for Factor Analysis.

In the first analysis, based on the Very Simple Structure criterion the analysis suggested a one-factor model, while a Parallel Analysis suggested a seven-factor model and an eigenvalue analysis suggested a six-factor model. Factor structures, beginning with one factor were examined based on the explained cumulative variances. The five-factor model proved best, as the cumulative variance for the six-factor model was 0.42 compared to 0.41 in the five-factor model. Next, items with lower than 0.3 factor loadings on any of the factors were discarded. As a Heywood case was detected with the five-factor model, the number of factors was decreased to four. For this four-factor structure (Table 3), model-fit indices were relatively low, but closer to the acceptable level than the other factor structures, $\chi_{\left(2\right)}^{2}$ = 8.48, *p* < 0.05, TLI = 0.82, RMSEA = 0.07 (95% CI [0.00, 0.13]).

**Table 3:** **Results of the exploratory factor analysis for the two tests**.

		**Factor 1**	**Factor 2**	**Factor 3**	**Factor 4**	**Communalities**
Test 1	Gambler's fallacy	0.00	−0.07	**0.53**	−0.06	0.27
	Sunk cost fallacy	0.15	−0.09	**0.37**	0.17	0.27
	Base-rate neglect	**0.98**	0.09	0.03	−0.01	0.99
	Monty Hall problem	−0.02	0.02	−0.03	**0.86**	0.73
	Insensitivity to sample size	**0.32**	−0.18	−0.06	−0.07	0.12
	Relativity bias	−0.06	0.09	**0.57**	−0.14	0.28
	Outcome bias	0.01	0.00	**0.47**	0.10	0.26
	Anchoring effect	0.09	**0.99**	0.00	0.01	0.99
Test 2	Gambler's fallacy	**0.90**	0.05	0.03	0.00	0.82
	Sunk cost fallacy	0.15	−0.10	0.16	0.24	0.15
	Base-rate neglect	0.10	−0.04	**0.51**	−0.08	0.26
	Monty Hall problem	0.05	**0.99**	−0.01	0.03	0.99
	Insensitivity to sample size	−0.10	−0.12	−0.01	0.17	0.05
	Relativity bias	−0.18	0.20	**0.40**	0.05	0.24
	Outcome bias	−0.01	0.04	−0.08	**0.68**	0.44
	Anchoring effect	−0.01	−0.11	**0.38**	−0.06	0.15

*Factor loadings of 0.3 and above are in bold font. Factors with one item loading are presented only for demonstrative purposes*.

In the Factor Analysis of the second test, we observed similar fitting indices, $\chi_{\left(2\right)}^{2}$ = 6.58, *p* < 0.05, TLI = 0.81, RMSEA = 0.05 (95% CI [0.00, 0.11]), however, no consistency was found between the factors of the two tests. As Table 3 indicates, the factor structures of the two tests varied greatly. These results suggest that the wording of the different reasoning problems affects how people interpret and answer the questions. Original item-item correlations and factor correlations can be found in the Supplementary Materials.

#### Correlation with the CRT

To further investigate the differences between the measured biases, for both tests the correlations between the CRT composite score and each HB task were assessed separately (Table 4). The average CRT performance was similar in the groups (*M*_*Test*1_ = 1.37; *M*_*Test*2_ = 1.27). The Fisher-exact test^3^ showed significantly different correlations in the two tests for Probability match, Base-rate neglect, Insensitivity to sample size, Monty Hall problem, and the Relativity bias.

**Table 4:** **Differences between the two tests in correlation with the CRT for each task**.

**Tasks**	**Correlation with the CRT composite**		**Fisher-exact test**
	***r* (Test 1)**	***r* (Test 2)**	***Z*-scores**
Anchoring effect	0.13^**^	0.004	1.85
Base-rate neglect	0.23^***^	−0.003	3.46^***^
Conjunction fallacy	−0.03	−0.10^*^	1.03
Covariation detection	0.05	−0.03	1.17
Framing effect	0.14^*^	0.05	1.32
Gambler's fallacy	0.11^*^	0.01	1.46
Insensitivity to sample size	0.11^*^	−0.04	2.19^*^
Monty Hall problem	0.19^***^	0.02	2.51^*^
Outcome bias	0.23^***^	0.16^**^	1.06
Probability match	0.11^*^	0.24^***^	−1.96*
Regression to the mean	−0.01	−0.04	0.44
Relativity bias	0.06	−0.09	2.19^*^
Sunk cost fallacy	0.08	0.14^**^	−0.89

* *p < 0.05*;

** *p < 0.01*;

*** *p < 0.001*.

#### Differences in accuracy between the HB tasks

To assess the degree to which the different wording of the tasks affected the participants' susceptibility on the biases, chi-square tests were conducted on performance scores. The results revealed significant differences in terms of accuracy for Anchoring effect, Conjunction fallacy, Covariation detection, Framing effect, Probability match, Regression to the mean, Relativity bias, Sunk cost fallacy and for the overall HB composite score (Table 5).

**Table 5:** **Differences in accuracy between the two tests**.

	**Test 1 (%)**	**Test 2 (%)**	$\chi_{\left(1\right)}^{2}$
Anchoring effect	49.27	76.41	65.61^***^
Base-rate neglect	41.26	35.90	2.41
Conjunction fallacy	31.99	8.21	76.02^***^
Covariation detection	23.90	16.98	6.49^**^
Framing effect	50.10	39.80	8.83^**^
Gambler's fallacy	34.92	30.98	1.89
Insensitivity to sample size	14.73	13.99	0.04
Monty Hall problem	23.97	22.08	0.33
Outcome bias	34.10	30.61	1.04
Probability match	22.10	43.56	50.85^***^
Regression to the mean	27.56	16.00	16.51^***^
Relativity bias	44.89	19.90	59.18^***^
Sunk cost fallacy	25.66	35.82	10.94^***^

*Accuracy reflects mean percentages of correct responses for each task*.

** *p < 0.01*;

*** *p < 0.001*.

### Discussion

The aim of this study was to explore the psychometric properties of two versions of a newly constructed HB questionnaire. The questionnaire contained modified versions of 13 frequently used HB tasks. Through these modifications we aimed to ascertain that all questions satisfy the criteria of comparability, construct validity, and motivation. To be able to assume that the tasks are equally sensitive for detecting the given biases, we unified them by providing only one correct option and three biased options for the participants to choose from. Choosing any of the three incorrect options should indicate failure to resist the susceptibility of the same bias or fallacy. To motivate them to care about their answers they had to envisage themselves in situations of a concrete decision maker making critical decisions. Although the effect of these modifications was not directly tested in this study, they serve as methodological recommendations for the development of multiple-bias questionnaires.

To study our main research question of whether different biases reflect the same individual differences factor, we analyzed the psychometric properties of the surveys. The reliability assessment of the composite scores on both of the two tests showed very low internal consistency. The indices would not allow the questionnaire to be used for measuring the same concept. Therefore, to explore the underlying factorial structure, we employed a polynomial Factor Analysis for both tests. For Test 1 a four-factor model fitted the data best, cumulatively explaining 49% of the variance. The same structure explained 39% of cumulative variance for Test 2. In both tests some HB tasks did not reach the expected level of loading. Most surprisingly, the explorations showed very different factor structure for the two tests. For example, the Anchoring Effect constituted a separate factor in Test 1, but it was grouped with Relativity bias and Base-rate neglect in Test 2, while these latter two were in separate factors in Test 1. In Test 1 five, and in Test 2 an additional three HB tasks did not reach the necessary loading for any of the factors.

Another way to assess the shared properties of these HB tasks is to correlate their scores with the CRT composite. The CRT was argued to be the most representative test of the assumed latent rationality factor (Toplak et al., 2011). The individual correlation coefficients of the tasks were within the range of -0.09 and 0.24 reaching the level of significance only occasionally. Importantly, in five cases these correlation coefficients were significantly different for the two tests, changing valence in three tasks.

These apparent differences between the two versions of the tasks call for explanation. One possibility is that while incorrect responses on the different versions constitute the violation of the same normative rule, different wordings of the tasks may evoke different strategies and may have a greater effect on performance than previously expected. We found support for the latter in the comparison of the accuracy measures of the tasks in the two tests. By altering the wording of the tasks, the Framing effect, Anchoring effect, Relativity bias, Probability match, Outcome bias, Covariation detection, Sunk cost fallacy, Conjunction fallacy, and Regression to the mean all showed significantly different levels of difficulty.

The other possible explanation for this pattern of results is that when we modified the original HB tasks, we unintentionally decreased the validity of the questions. Although in devising these new tasks, we always aimed to keep the structure of the original tasks while changing the number of answer options and some superficial features of the tasks, some modifications might have decreased the ability of the test to measure the cognitive processes that underlie the original tasks. A limitation of this study is that we did not administer the original questions, and therefore, this assumption could not be tested directly. Study 2 was designed to explore this second possible explanation of our present results.

## Study 2

In this study, for each of seven frequently tested cognitive biases we collected three different tasks. The different versions of the tasks have been used interchangeably in the literature to measure the given cognitive bias. We assumed that if different test questions of a bias measure the same underlying factor, then the test questions are interchangeable. We created three questionnaires for the seven biases by randomly selecting one version of each task for each questionnaire. If the different wording of the task does not alter the cognitive strategy that the participant employs when solving the task then we would expect the same pattern of factors emerging from the Factor Analysis. Also, if the different versions of the task represent the same underlying factor, then we would expect that they would correlate similarly with the CRT.

### Methods

#### Participants

Five hundred and twenty seven native English-speaking participants (277 female, *M* = 37.98 years, *SD* = 12.18) were recruited through an online crowd-sourcing platform, CrowdFlower. In exchange for their participation, they were paid 0.30 USD for finishing the questionnaire. The research was approved by the institutional ethics committee of Eotvos Lorand University, Hungary.

#### Materials

Three new HB questionnaires were constructed, each consisting of seven tasks (Base-rate neglect, Conjunction fallacy, Covariation detection, Framing effect, Gambler's fallacy, Insensitivity to sample size, Sunk cost fallacy). Three versions of each task were collected from the literature and were randomly assigned to the tests (Supplementary Material, Study 2). All testing materials were presented in English. For each task, the aim was to employ questions with the same number of answer options. Where this was not possible, the number of answer options was modified to render them more similar to the other tasks. Similarly to Study 1, the CRT tasks were administered at the end of the survey.

#### Procedure

Participants were randomly assigned to one of the three tests. After giving informed consent, participants were asked to solve the seven HB tasks and the three CRT questions (Frederick, 2005). The first part of the framing question was administered at the beginning of the test, followed by the six other HB questions in randomized order. The second part of the risky-choice framing question was administered after the other HB questions and before the CRT tasks. No time-pressure was employed in this study.

### Results

#### Reliability

Reliability measures showed weaker results for the composite scores than in Study 1. Both Split-half and Cronbach's alpha values were below the acceptable level (Table 6).

**Table 6:** **Reliability measures for the composite score of the three HB tests**.

	**Split-half**	**Cronbach's alpha**
Test 1	0.35	0.08
Test 2	0.37	0.16
Test 3	0.22	−0.004

#### Factor analysis

An explorative Factor Analysis, similar to Study 1, was conducted on the three tests. Prior to the Factor Analysis, data adequacy was tested identically to Study 1. With regard to Test 1, the KMO reached an acceptable level, KMO = 0.59, and the Bartlett's test was significant again, $\chi_{\left(21\right)}^{2}$ = 159.54, *p* < 0.001. For Test 2 similar results were obtained: KMO = 0.65, Bartlett's test, $\chi_{\left(21\right)}^{2}$ = 152.92, *p* < 0.0001. In Test 3, the KMO was smaller, KMO = 0.33, but Bartlett's test was significant, $\chi_{\left(21\right)}^{2}$ = 250.69, *p* < 0.001. Contrary to the small KMO value in Test 3, the mean absolute correlation was relatively high, *r* = 0.3. These results suggested that a Factor Analysis can be conducted on the data.

In the first analysis, based on the Very Simple Structure criterion the analysis suggested a three-factor model, while a Parallel Analysis suggested a two-factor model and an eigenvalue analysis suggested a three-factor model. Based on explained cumulative variance, the three-factor structure fitted the data best for all of the three tests. Model-fit indices were acceptable for Test 1, $\chi_{\left(3\right)}^{2}$ = 2.04, *p* = 0.56, TLI = 1.00, RMSEA = 0.00 (95% CI [0.00, 0.11]), and Test 2, $\chi_{\left(3\right)}^{2}$ = 3.1, *p* = 0.38, TLI = 1.00, RMSEA = 0.02 (95% CI [0.00, 0.14]), but they were not acceptable for Test 3^4^, $\chi_{\left(3\right)}^{2}$ = 22.57, *p* < 0.05, TLI = 0.33, RMSEA = 0.21 (95% CI [0.12, 0.29]). Similarly to Study 1, different tasks belonged to different factors in each test, for example, Base-rate neglect was classified in one factor with Insensitivity to sample size, Gambler's fallacy and Sunk cost fallacy in Test 1, with Conjunction fallacy in Test 2, and with Sunk cost fallacy in Test 3 (Table 7). Original item-item correlations and factor correlations can be found in the Supplementary Materials.

**Table 7:** **Results of the exploratory factor analysis for the three tests**.

		**Factor 1**	**Factor 2**	**Factor 3**	**Communalities**
Test 1	Conjunction fallacy	0.03	0.03	−**0.49**	0.24
	Base-rate neglect	**0.48**	−0.08	0.19	0.3
	Covariation detection	0.07	0.03	**0.69**	0.5
	Insensitivity to sample size	−**0.48**	0.07	0.16	0.22
	Gambler's fallacy	**0.94**	0.06	0.05	0.93
	Framing effect	0.08	**0.99**	0.02	0.99
	Sunk cost fallacy	**0.30**	0.04	−0.06	0.09
Test 2	Conjunction fallacy	−0.24	0.06	**0.5**	0.18
	Base-rate neglect	−0.04	−0.03	**0.54**	0.26
	Covariation detection	**0.59**	−0.23	0.08	0.45
	Insensitivity to sample size	−**0.42**	−0.1	−0.13	0.27
	Gambler's fallacy	**0.85**	0.12	−0.17	0.6
	Framing effect	0.04	**0.99**	0.02	0.99
	Sunk cost fallacy	0.3	0.03	0.35	0.33
Test 3	Conjunction fallacy	0.46	−0.19	0.48	0.57
	Base-rate neglect	−0.46	0.16	**0.98**	0.99
	Covariation detection	−0.02	0.09	0.15	0.03
	Insensitivity to sample size	**0.84**	−0.02	0.13	0.69
	Gambler's fallacy	**0.55**	−0.28	0.13	0.39
	Framing effect	−0.06	**1.00**	0.11	0.99
	Sunk cost fallacy	0.05	0.07	−**0.39**	0.18

*Factor loadings of 0.3 and above are marked with bold. Factors with one item loading are presented only for demonstrative purposes*.

#### Correlations with the CRT composite score

Similarly to Study 1, the correlation between the HB tasks and the CRT composite scores were examined; the results are presented in Tables 8, 9. No significant correlation between the Framing effect and the CRT composite scores were found (Table 8). The Fisher-exact test revealed differing levels of correlation for the Conjunction fallacy, for the Insensitivity to sample size and for the Sunk cost fallacy (Table 9). Average CRT performance was similar in each group (*M*_Test1_ = 1.31; *M*_Test2_ = 1.27; *M*_Test3_ = 1.40).

**Table 8:** **Correlations of each HB tasks with the CRT composite scores**.

	**Test 1**	**Test 2**	**Test 3**
Base-rate neglect	0.22^*^	0.26^***^	0.28^***^
Conjunction fallacy	−0.16^*^	0.08	0.19^*^
Covariation detection	0.32^***^	0.34^***^	0.15^*^
Framing effect	0.08	0.06	−0.08
Gamblers fallacy	0.26^***^	0.21^*^	0.18^*^
Insensitivity to sample size	0.05	−0.34^***^	0.04
Sunk cost fallacy	0.09	0.23^*^	−0.18^*^

*** *p < 0.001*;

* *p < 0.05*.

**Table 9:** **Differences in correlation between the tests for each HB Task**.

	**Test 1–Test 2**	**Test 1–Test 3**	**Test 2–Test 3**
Base-rate neglect	−0.40	−0.60	−0.20
Conjunction fallacy	−2.25^*^	−3.30^***^	−1.04
Covariation detection	−0.21	1.69	1.89
Framing	0.19	1.50	1.31
Gamblers fallacy	0.49	0.79	0.29
Insensitivity to sample size	3.77^***^	−0.38	−4.14^***^
Sunk cost	−1.34	2.54^*^	3.88^***^

*Presented values represent Z-scores*.

* *p < 0.05*;

*** *p < 0.001*.

#### Differences in accuracy between the HB tasks

As in Study 1, chi-square tests were conducted on performance scores to examine the differences between the different tests. The analyses revealed significant differences in terms of accuracy across the three versions of the HB task, except for Gambler's fallacy (Table 10).

**Table 10:** **Accuracy for each task and composite score across the three HB tests**.

**Tasks**	**Test 1 (%)**	**Test 2 (%)**	**Test 3 (%)**	$\chi_{\left(2,\;528\right)}^{2}$
Base-rate neglect	39.33	46.02	58.76	13.85^***^
Conjunction fallacy	34.27	9.66	24.86	30.82^***^
Covariation detection	38.20	56.82	31.64	24.68^***^
Framing effect	73.60	76.70	53.11	26.63^***^
Gambler's fallacy	85.96	89.20	92.66	4.16
Insensitivity to sample size	27.53	17.05	81.92	175.87^***^
Sunk cost fallacy	61.80	75.00	29.94	76.78^***^

*Accuracy reflects mean percentages of correct responses for each task*.

*** *p < 0.001*.

### Discussion

The aim of this study was to assess the properties of three versions of an HB task questionnaire. We assumed that if different test questions of a bias measure the same underlying factor, then the test questions are interchangeable. We thereby expected the same pattern of factors emerging from the Factor Analysis and similar correlation between the tasks and the CRT.

The reliability measures of the three tests were very poor, arguing against a general factor behind the HB tasks. The Factor Analysis showed a no more coherent picture of the tasks here than in Study 1. For example, in Test 1, Base-rate neglect was grouped in one factor with Sunk Cost and Gambler's fallacy, with negative loading of Insensitivity to sample size. In Test 2 Base-rate neglect was in the same factor with Conjunction fallacy, while in Test 3 Base-rate neglect was paired with the Sunk cost fallacy. Only the Insensitivity to sample size and the Gambler's fallacy tasks belong to the same factor in all three questionnaires. The three versions of the tasks showed significantly different correlations with the CRT for the Conjunction fallacy, the Insensitivity to sample size and the Sunk cost tasks. Scores of the Framing effect task did not show significant correlation with the CRT in any of the tests. The susceptibility of the tasks also greatly varies among the tests, showing similarly good performance only for the Gambler's fallacy task, for the other tests the accuracy measure significantly differed. These results shed light on a general problem with the HB tasks that might explain the unexpected findings of our Study 1.

## General discussion

The objective of this paper is to highlight certain methodological questions in individual differences research of cognitive biases. In Study 1, we emphasized that the within-subjects design, which is required for measuring individual differences, brings about new challenges when multiple-bias surveys are created. The problem of turning between-subjects tasks into within-subjects tasks is especially problematic for tests of coherence rationality (the expectation that the person should decide indifferently in logically equivalent situations; Kahneman and Fredrick, 2005) such as the framing effect, as the expected behavior can become transparent to the participants. We also stressed the importance of other criteria of this design, such as comparability, construct validity, and motivation. We implemented the necessary modifications on the traditionally used HB tasks to satisfy these criteria. Participants have been found to be susceptible to the questions of the two new surveys. Nevertheless, the results of Factor Analysis indicated major inconsistencies between the two tests of the same biases. In Study 2, where we returned to using the traditional versions of the HB tasks, this inconsistency remained apparent among the three questionnaires. It appeared that using different versions of the HB tasks resulted in remarkably different factor structures, altering correlational relations with the CRT and varying bias-susceptibility. These results raise several important questions for the research of individual differences in cognitive biases.

### What does the HB composite score represent?

With a few exceptions (e.g., Bruine de Bruin et al., 2007; Klaczynski, 2014), most multiple-bias questionnaires consist of one or two task items from each measured bias (e.g., Slugoski et al., 1993; Stanovich and West, 1998; Klaczynski, 2001; Toplak et al., 2007, 2011; West et al., 2008, 2012). Performance scores on these items are regularly aggregated to create a composite score for further analyses. It is not clear, however, what we expect this composite score to represent. The view that there is a general underlying factor behind HB is rarely held. Earlier proposals for one single factor behind the diverse set of decision errors have been empirically discouraged. For example, Wyer and Srull (1989) claimed that the variety of decision and judgment biases is the result of people's general tendency to treat conditional relationships as if they were biconditional (the disposition to infer “Y is X” given “X is Y”). Assuming individual difference variation in performance, we would expect the HB to show high intercorrelation and to load highly on one factor. Factor Analysis did not support Wyer and Srull's proposal (Slugoski et al., 1993). Studies of decision making competence found more promising rates of explained variance by one-factor models (30% for Bruine de Bruin et al., 2007; 25% for Parker and Fischhoff, 2005). However, the components of their test batteries (Resistance to Framing, Recognizing Social Norms, Under/overconfidence, Applying Decision Rules, Consistency in Risk Perception, Path Independence, and Resistance to Sunk Costs) only partially represent the traditional HB tasks. Intercorrelations on different sets of tasks were repeatedly found to be weak (0.16 for Bruine de Bruin et al., 2007; 0.12 for Parker and Fischhoff, 2005), occasionally being very weak (e.g., 0.066 for West et al., 2008; 0.03 for Slugoski et al., 1993). While the unidimensional factor bears no theoretical or empirical support, research literature shows several attempts to classify decision biases into sets of similar problems. When the researchers choose their test questions they often assume that some combination of these tasks measure probabilistic reasoning abilities (Toplak et al., 2007), the conceptual measure of rational thought (Stanovich et al., 2011), or decision competence (Bruine de Bruin et al., 2007). The most elaborated bias taxonomy was provided by Stanovich (2009, 2012). There, it is suggested that rational thinking problems can be classified according to three main categories of cognitive difficulties: the cognitive miser problem such as focal bias, override failure; mindware gaps, such as probability knowledge or alternative thinking; and the contaminated mindware such as self and egocentric processing. Stanovich suggests that errors on most of the HB tasks can be linked to one of these categories, while some biases are determined by multiple cognitive problems. It is proposed that these dimensions of rationality can be measured by specific traditional testing paradigms. From a testing perspective, Stanovich relies on the psychometric separability of these tasks to allow for a “Rationality Quotient,” a comprehensive assessment of rational thought (2011). This proposal strongly depends on the assumptions that the traditionally used HB tasks can be grouped by their underlying factors, and that different versions of the same task represent the same thinking error. Some evidence for the separability of biases come from the study of Toplak et al. (2007) where in a communality analysis of Gambling fallacy, Regression to the mean, Covariation detection, Probability matching, Bayesian reasoning, Statistical reasoning, Outcome bias, and Probability reasoning tasks they found that three categories of these tasks explained unique variance in problem gambling. Although Factor Analysis has been applied to decision bias collections, their direct aim was not exploration of such classifications (Klaczynski, 2001; Parker and Fischhoff, 2005; Bruine de Bruin et al., 2007). The recent work of Teovanović et al. (2015) has been dedicated to the examination of factorial structure on HB tasks. Performance on the Anchoring effect, Belief bias, Overconfidence bias, Hindsight bias, Base-rate neglect, Outcome bias and Sunk cost effect has been analyzed in an Exploratory Factor Analysis. They argued that their results showed low explained variance, indicating weak replicability. When they analyzed these tasks together with cognitive ability tests and thinking disposition measures in another Factor Analysis, the previously observed factors were not replicated. These findings, in accord with our own present results, indicate that contrary to previous theoretical expectations, decision biases do not form robust categories, or at least they cannot be extracted by the traditionally used HB tasks. These results question the empirical grounding that HB composite scores provide a meaningful measure for the exploration of individual differences in decision competence.

### What do the different versions of an HB task measure?

Besides the surprisingly low communality of the HB tasks, the present work points to an additional concern: the observation that the different versions of the HB tasks appear to show an unexpected level of heterogeneity. In fact, in most previous studies when several tasks of the same bias have been assessed together, people's susceptibility to the different versions of the task greatly varied (e.g., West et al., 2008) and internal consistency of these tasks rarely reached acceptable levels (0.61 for Resistance to Sunk Cost in Bruine de Bruin et al., 2007; yet the same bias is 0.03 in Parker and Fischhoff, 2005). Measuring the different items of the same task in within-subjects arrangement can increase intercorrelation only by sequential effects or as a result of the participants' desire to appear to be consistent with their answers to recognizably similar questions (coherence rationality, Kahneman and Fredrick, 2005). When we found inconsistencies between the different versions of the tasks, we used between-subjects comparisons. This form of analysis is necessary in order to understand the idiosyncratic properties of the tasks, since a participant's answer on one cannot influence their answer on the other task. Although the degree of intercorrelation is not a direct indicator of whether the items measure a unidimensional latent construct (Green et al., 1977), the result that different items of a task fall into different factors strongly suggests that they either do not measure the same latent construct, or that individual items have very poor measurement properties.

### Possible causes for the inconsistencies

At this point, it is only within the realm of speculation to suggest an explanation for this inconsistency among HB task versions. One possibility is that people interpret the questions differently than how the questions were intended by the researchers. Similarly to the studies of the framing effect, a vast body of research in survey studies (e.g., Bradburn, 1982; Schwarz, 1999) suggests that seemingly irrelevant wording of the questions and response options can have a significant effect on people's tendency to interpret and answer the questions. For example, when researchers use presumed antonyms (e.g., “forbidding” and “allowing”), participants may not treat these terms as exact opposites (Rugg, 1941). Synonymous terms (e.g., “inflation” and “prices in general”), may be interpreted differently (Ranyard et al., 2008) due to the difference between lexical and pragmatic meanings (Schwarz, 1999). Varying cover stories and the employed situational factors can easily lead to a mismatch between how the researcher and the participant interpret the question.

From a contextualist perspective, problems framed in different domains might evoke different cognitive strategies (Cosmides and Tooby, 1989; Gigerenzer and Hug, 1992). It is reasonable to assume that people show idiosyncrasy in their approach to a problem under the influence of prior knowledge or domain familiarity. For example, it has been found that people perform generally better on versions of the Wason Selection Task when presented in the context of social relations (Cosmides, 1989). Similarly, studies of risk-perception and decision making competence showed differences between domains of financial decisions, health/safety, recreational, ethical, and social decisions (Weber et al., 2002; Weller et al., 2015). It has been suggested that task content can elicit different decision mode usage (analytic, rule-based, automatic) and these modes can lead to different degrees of bias susceptibility (Blais and Weber, 2001), and also that changes in content can change decision outcome by affecting strategies and mental representations (Rettinger and Hastie, 2001). Jackson et al. (2015, submitted) have demonstrated that context is important for adjusting individuals' control thresholds, which in turn affect their recklessness and hesitancy. Therefore, the domain-specificity framework would predict inconsistencies in the different versions of tasks if they are framed in different domains as they can require different cognitive strategies and abilities.

The format of response options can also inadvertently bias the measurement of the task items. In HB literature, response options are presented in very different formats, such as open-ended questions (e.g., Toplak et al., 2011), simple choice (e.g., Klaczynski, 2001), multiple choice (e.g., Kahneman et al., 1982), or rating scales (e.g., Bruine de Bruin et al., 2007). Response mode can greatly affect people's performance (de Bruin and Fischhoff, 2000; Roediger and Marsh, 2005), confidence (Pallier et al., 2002; Jackson, submitted), and the cognitive strategies elicited (Hertwig and Chase, 1998).

The greatest difficulty in this matter is that we know very little about the underlying cognitive processes of the different HB. On the one hand, it has been shown at least for the anchoring heuristic (Epley and Gilovich, 2005) and the framing effect (Levin et al., 1998, 2002) that these are labels representing a variety of different cognitive mechanisms dependent on question content and task characteristics. On the other hand, a repeated critique of the heuristics-and-biases approach is that its labels are either so vaguely defined that they do not allow falsifiable process models (Gigerenzer and Goldstein, 1996) or the attempts to explore the underlying processes in judgment and reasoning are unsatisfying (Fiedler, 2015; Fiedler and von Sydow, 2015).

Another possible source of inconsistency between the different versions of the tasks is that they tap into different normative models. Many critics have insisted that on certain tasks the responses judged to be wrong by the researcher are in fact correct due to the mismatch between the problem and the linked normative model (Margolis, 1987; Messer and Griggs, 2013). It has been argued that taking different linguistic or conceptual interpretations of the problems may to lead to different normative answers for tasks such as the taxicab base-rate problem (Birnbaum, 1983), the overconfidence effect (Gigerenzer, 1991), or the conjunction fallacy task (Fiedler, 1988). Therefore, it is possible that different cover stories or different wording of the tasks inadvertently change the corresponding normative models, which might require different competence from the participant.

Even if we suppose that errors on task versions of the same cognitive bias are caused by the same cognitive failure, different levels of task fluency can alter the heuristic nature of the task. On certain tasks, for example, where higher level of fluency triggers a stronger heuristic answer (Thompson et al., 2011), good performance might indicate higher reflectivity, while tasks with a lower level of fluency do not require the inhibition of the immediate answer and rather reflect people's cognitive capacity. This notion is confirmed by the significantly different correlation coefficients among the tasks of a given bias and the CRT composite scores. Arguably, the CRT measures inhibition or reflectivity (Campitelli and Labollita, 2010), and thus differences among correlation coefficients could be caused by the different level of reflectivity needed to solve the tasks. Our results, with the support of the empirical studies outlined here, may therefore suggest that the diverse collection of questions traditionally used for measuring the individual decision biases cannot be taken unconditionally as interchangeable measures of the same latent factor or cognitive mechanism. Rather, more effort is needed to explore how content, language and question format can alter or influence the assessment of a decision bias.

## Conclusions

In summary, this paper represents two attempts to explore the methodological requirements for individual differences research using HB tasks. The first study highlighted the need to construct HB test items that satisfy the criteria of comparability, construct validity, and motivation. However, the results of a modified HB test battery suggested and the analysis of the second study confirmed a great level of inconsistency when these biases are measured by individual items. An overview of decision competence literature suggests that the weakness of these tests cannot be derived from the general practice of measuring biases by single (or very few) items for two reasons. Firstly, multiple-bias questionnaires show poor or unacceptable internal consistency. Secondly, the empirical results do not support the theoretical assumption that the different versions of the HB tasks measure the same underlying cognitive construct. While in the field of judgment and decision making there is a growing interest in going beyond aggregate level results by examining individual differences, the success will depend on how clearly we can understand the cognitive mechanisms behind the traditional list of HB.

### Conflict of interest statement

The authors declare that the research was conducted in the absence of any commercial or financial relationships that could be construed as a potential conflict of interest.

## Footnotes

^1^The participant's results were excluded from the analysis if their CRT answers clearly indicated that the participant was not motivated to answer sensibly. Practically, for the Bat and Ball problem of the CRT task, we excluded those participants who provided nonsensical answers (such as 55,555, 12,345, or 10,000,000) and retained those who gave the correct, intuitive incorrect answers or anything that an imaginable miscalculation could yield.

^2^Additionally, an Overconfidence bias and a Planning fallacy task were administered, but as they did not satisfy the methodological criteria of having a 25% chance-level of giving a correct answer, they were not included in the analyses.

^3^Correlation coefficients were compared using a Fisher *r*-to-*z* transformation (http://vassarstats.net/rdiff.html).

^4^Note that the factor structure in Table 7 of Test 3 is only presented for demonstrational purposes. These results are not meaningful due to the low fitting indices, which suggest that the performance scores on Test 3 are not suitable to extract latent factors from.

## References

[B1] Bar-Hillel M. (1980). The base-rate fallacy in probability judgments. Acta Psychol. (Amst). 44, 211–233. 10.1016/0001-6918(80)90046-3

[B2] Birnbaum M. H. (1983). Base rates in bayesian inference: signal detection analysis of the cab problem. Am. J. Psychol. 96, 85–94. 10.2307/1422211

[B3] Blais A.-R. Weber E. U. (2001). Domain-specificity and gender differences in decision making. Risk Decis. Policy 6, 47–69. 10.1017/S1357530901000254

[B4] Bradburn N. (1982). Question-wording effects in surveys, in Question Framing and Response Consistency, ed Hogarth R. M. (San Francisco, CA: Jossey-Bass), 65–76.

[B5] Bruine de Bruin W. Parker A. M. Fischhoff B. (2007). Individual differences in adult decision-making competence. J. Pers. Soc. Psychol. 92, 938–956. 10.1037/0022-3514.92.5.938 17484614

[B6] Camerer C. F. Hogarth R. M. (1999). The effects of financial incentives in experiments: a review and capital-labor-production framework. J. Risk Uncertain. 19, 7–42. 10.1023/A:1007850605129

[B7] Campitelli G. Labollita M. (2010). Correlations of cognitive reflection with judgments and choices. Judgm. Decis. Mak. 5, 182–191.

[B8] Christensen-Szalanski J. J. Willham C. F. (1991). The hindsight bias: a meta-analysis. Organ. Behav. Hum. Decis. Process. 48, 147–168. 10.1016/0749-5978(91)90010-Q

[B9] Cosmides L. (1989). The logic of social exchange: has natural selection shaped how humans reason? Studies with the Wason selection task. Cognition 31, 187–276. 10.1016/0010-0277(89)90023-1 2743748

[B10] Cosmides L. Tooby J. (1989). Evolutionary psychology and the generation of culture, part II: case study: a computational theory of social exchange. Ethol. Sociobiol. 10, 51–97. 10.1016/0162-3095(89)90013-7

[B11] de Bruin W. B. Fischhoff B. (2000). The effect of question format on measured HIV/AIDS knowledge: detention center teens, high school students, and adults. AIDS Educ. Prev. 12, 187–198. 10926123

[B12] Epley N. Gilovich T. (2005). When effortful thinking influences judgmental anchoring: differential effects of forewarning and incentives on self−generated and externally provided anchors. J. Behav. Decis. Mak. 18, 199–212. 10.1002/bdm.495

[B13] Evans J. S. B. Stanovich K. E. (2013). Dual-process theories of higher cognition advancing the debate. Perspect. Psychol. Sci. 8, 223–241. 10.1177/1745691612460685 26172965

[B14] Fiedler K. (1988). The dependence of the conjunction fallacy on subtle linguistic factors. Psychol. Res. 50, 123–129. 10.1007/BF00309212

[B15] Fiedler K. (2015). Functional research and cognitive-process research in behavioural science: an unequal but firmly connected pair. Int. J. Psychol. 10.1002/ijop.12163. [Epub ahead of print]. 25921294

[B16] Fiedler K. von Sydow M. (2015). Heuristics and biases: beyond Tversky and Kahneman's (1974) judgment under uncertainty, in Cognitive Psychology: Revisiting the Classical Studies, eds Eysenck M. W. Groome D. (Los Angeles, US: Sage), 146–161.

[B17] Frederick S. (2005). Cognitive reflection and decision making. J. Econ. Perspect. 19, 25–42. 10.1257/089533005775196732

[B18] Frisch D. (1993). Reasons for framing effects. Organ. Behav. Hum. Decis. Process. 54, 399–429. 10.1006/obhd.1993.1017

[B19] Gambara H. Piñon A. (2005). A meta-analytic review of framing effect: risky, attribute and goal framing. Psicothema 17, 325–331.

[B20] Gigerenzer G. (1991). How to make cognitive illusions disappear: beyond “Heuristics and Biases.” Eur. Rev. Soc. Psychol. 2, 83–115. 10.1080/14792779143000033

[B21] Gigerenzer G. Goldstein D. G. (1996). Reasoning the fast and frugal way: models of bounded rationality. Psychol. Rev. 103, 650–669. 10.1037/0033-295X.103.4.650 8888650

[B22] Gigerenzer G. Hug K. (1992). Domain-specific reasoning: social contracts, cheating, and perspective change. Cognition 43, 127–171. 10.1016/0010-0277(92)90060-U 1617917

[B23] Gigerenzer G. Todd P. M. Group A. R. (1999). Simple Heuristics that Make us Smart. New York, NY: Oxford University Press.

[B24] Gilovich T. Vallone R. Tversky A. (1985). The hot hand in basketball: on the misperception of random sequences. Cogn. Psychol. 17, 295–314. 10.1016/0010-0285(85)90010-6

[B25] Green S. B. Lissitz R. W. Mulaik S. A. (1977). Limitations of coefficient alpha as an index of test unidimensionality1. Educ. Psychol. Meas. 37, 827–838. 10.1177/001316447703700403

[B26] Hardt O. Pohl R. F. (2003). Hindsight bias as a function of anchor distance and anchor plausibility. Memory 11, 379–394. 10.1080/09658210244000504 14562869

[B27] Hertwig R. Chase V. (1998). Many reasons or just one: how response mode affects reasoning in the conjunction problem. Think. Reason. 4, 319–352. 10.1080/135467898394102

[B29] Jackson S. A. Kleitman S. Stankov L. Howie P. (2015). Decision pattern analysis as a general framework for studying individual differences in decision making. J. Behav. Decis. Mak. 10.1002/bdm.1887. [Epub ahead of print].

[B31] Kahneman D. Fredrick S. (2005). A model of heuristic judgment, in The Cambridge Handbook of Thinking and Reasoning, eds Holyoak K. J. Morrison R. G. (New York, NY: Cambridge University Press), 267–293.

[B32] Kahneman D. Slovic P. Tversky A. (1982). Judgment Under Uncertainty: Heuristics and Biases. Cambridge, UK: Cambridge University Press 10.1017/CBO9780511809477 17835457

[B33] Klaczynski P. A. (2001). Analytic and heuristic processing influences on adolescent reasoning and decision−making. Child Dev. 72, 844–861. 10.1111/1467-8624.00319 11405586

[B34] Klaczynski P. A. (2014). Heuristics and biases: interactions among numeracy, ability, and reflectiveness predict normative responding. Front. Psychol. 5:665. 10.3389/fpsyg.2014.00665 25071639 PMC4078194

[B35] Klein G. A. (1999). Sources of Power: How People Make Decisions. Cambridge, MA: MIT press.

[B36] LeBoeuf R. A. Shafir E. (2003). Deep thoughts and shallow frames: on the susceptibility to framing effects. J. Behav. Decis. Mak. 16, 77–92. 10.1002/bdm.433

[B37] Levin I. P. Gaeth G. J. Schreiber J. Lauriola M. (2002). A new look at framing effects: distribution of effect sizes, individual differences, and independence of types of effects. Organ. Behav. Hum. Decis. Process. 88, 411–429. 10.1006/obhd.2001.2983

[B38] Levin I. P. Schneider S. L. Gaeth G. J. (1998). All frames are not created equal: a typology and critical analysis of framing effects. Organ. Behav. Hum. Decis. Process. 76, 149–188. 10.1006/obhd.1998.2804 9831520

[B39] Lichtenstein S. Fischhoff B. (1977). Do those who know more also know more about how much they know? Organ. Behav. Hum. Perform. 20, 159–183. 10.1016/0030-5073(77)90001-0

[B40] Margolis H. (1987). Patterns, Thinking, and Cognition: A Theory of Judgment. Chicago, IL: University of Chicago Press.

[B41] Messer W. S. Griggs R. A. (2013). Another look at Linda. Bull. Psychon. Soc. 31, 193–196. 10.3758/BF03337322

[B42] Pallier G. Wilkinson R. Danthiir V. Kleitman S. Knezevic G. Stankov L.. (2002). The role of individual differences in the accuracy of confidence judgments. J. Gen. Psychol. 129, 257–299. 10.1080/00221300209602099 12224810

[B43] Parker A. M. Fischhoff B. (2005). Decision−making competence: external validation through an individual−differences approach. J. Behav. Decis. Mak. 18, 1–27. 10.1002/bdm.481

[B44] Ranyard R. Missier F. D. Bonini N. Duxbury D. Summers B. (2008). Perceptions and expectations of price changes and inflation: a review and conceptual framework. J. Econ. Psychol. 29, 378–400. 10.1016/j.joep.2008.07.002

[B45] Rettinger D. A. Hastie R. (2001). Content effects on decision making. Organ. Behav. Hum. Decis. Process. 85, 336–359. 10.1006/obhd.2000.2948 11461205

[B46] Revelle W. (2014). psych: Procedures for Personality and Psychological Research. R package version, 1.5.4. Evanston: Northwestern University.

[B47] Roca M. Hogarth R. M. Maule A. J. (2006). Ambiguity seeking as a result of the status quo bias. J. Risk Uncertain. 32, 175–194. 10.1007/s11166-006-9518-8

[B48] Roediger H. L. III Marsh E. J. (2005). The positive and negative consequences of multiple-choice testing. J. Exp. Psychol. Learn. Mem. Cogn. 31, 1155–1159. 10.1037/0278-7393.31.5.1155 16248758

[B49] Rugg D. (1941). Experiments in wording questions: II. Public Opin. Q. 5, 91 10.1086/265467

[B50] Samuelson W. Zeckhauser R. (1988). Status quo bias in decision making. J. Risk Uncertain. 1, 7–59. 10.1007/BF00055564

[B51] Schwarz N. (1999). Self-reports: how the questions shape the answers. Am. Psychol. 54, 93–105. 10.1037/0003-066X.54.2.93

[B52] Simon H. A. (1955). A behavioral model of rational choice. Q. J. Econ. 69, 99–118. 10.2307/1884852

[B53] Slugoski B. R. Shields H. A. Dawson K. A. (1993). Relation of conditional reasoning to heuristic processing. Pers. Soc. Psychol. Bull. 19, 158–166. 10.1177/0146167293192004

[B54] Stanovich K. E. (1999). Who is Rational? Studies of Individual Differences in Reasoning. Hillsdale, NJ: Lawrence Erlbaum.

[B55] Stanovich K. E. (2009). What Intelligence Tests Miss: The Psychology of Rational Thought. New Haven, CT: Yale University Press.

[B56] Stanovich K. E. (2012). On the distinction between rationality and intelligence: implications for understanding individual differences in reasoning, in The Oxford Handbook of Thinking and Reasoning, eds Holyoak K. J. Morrison R. G. (New York, NY: Oxford University Press), 343–365. 10.1093/oxfordhb/9780199734689.013.0022

[B57] Stanovich K. E. West R. F. (1998). Individual differences in rational thought. J. Exp. Psychol. Gen. 127, 161–188. 10.1037/0096-3445.127.2.161

[B58] Stanovich K. E. West R. F. (2001). Individual differences in reasoning: implications for the rationality debate? Behav. Brain Sci. 23, 645–665. 10.1017/S0140525X00003435 11301544

[B59] Stanovich K. E. West R. F. Toplak M. E. (2011). Intelligence and rationality, in The Cambridge Handbook of Intelligence, eds Sternberg R. J. Kaufman S. B. (Cambridge, UK: Cambridge University Press), 784–826. 10.1017/CBO9780511977244.040

[B60] Teovanović P. Knežević G. Stankov L. (2015). Individual differences in cognitive biases: evidence against one-factor theory of rationality. Intelligence 50, 75–86. 10.1016/j.intell.2015.02.008

[B61] Thompson V. A. Prowse Turner J. A. Pennycook G. (2011). Intuition, reason, and metacognition. Cogn. Psychol. 63, 107–140. 10.1016/j.cogpsych.2011.06.001 21798215

[B62] Toplak M. E. Liu E. MacPherson R. Toneatto T. Stanovich K. E. (2007). The reasoning skills and thinking dispositions of problem gamblers: a dual−process taxonomy. J. Behav. Decis. Mak. 20, 103–124. 10.1002/bdm.544

[B63] Toplak M. E. West R. F. Stanovich K. E. (2011). The Cognitive Reflection Test as a predictor of performance on heuristics-and-biases tasks. Mem. Cognit. 39, 1275–1289. 10.3758/s13421-011-0104-1 21541821

[B64] Toplak M. E. West R. F. Stanovich K. E. (2014). Rational thinking and cognitive sophistication: development, cognitive abilities, and thinking dispositions. Dev. Psychol. 50, 1037–1048. 10.1037/a0034910 24188038

[B65] Tversky A. Kahneman D. (1983). Extensional versus intuitive reasoning: the conjunction fallacy in probability judgment. Psychol. Rev. 90, 293–315. 10.1037/0033-295X.90.4.293 PMC4658440 26635712

[B66] Tversky A. Kahneman D. (1986). Rational choice and the framing of decisions. J. Bus. 59, 251–278. 10.1086/296365

[B67] Weber E. U. Blais A. R. Betz N. E. (2002). A domain-specific risk-attitude scale: measuring risk perceptions and risk behaviors. J. Behav. Decis. Mak. 15, 263–290. 10.1002/bdm.414

[B68] Weller J. A. Ceschi A. Randolph C. (2015). Decision-making competence predicts domain-specific risk attitudes. Front. Psychol. 6:540. 10.3389/fpsyg.2015.00540 PMC4428222 26029128

[B69] West R. F. Meserve R. J. Stanovich K. E. (2012). Cognitive sophistication does not attenuate the bias blind spot. J. Pers. Soc. Psychol. 103, 506–519. 10.1037/a0028857 22663351

[B70] West R. F. Toplak M. E. Stanovich K. E. (2008). Heuristics and biases as measures of critical thinking: associations with cognitive ability and thinking dispositions. J. Educ. Psychol. 100, 930–941. 10.1037/a0012842

[B71] Wyer R. S. Jr. Srull T. K. (1989). Memory and Cognition in its Social Context. Hillsdale, NJ: Lawrence Erlbaum.

