# Supplementary material for: Measuring Individual Differences in Decision Biases: Methodological Considerations
Source: Front Psychol. 2015 Nov 19;6:1770. doi: 10.3389/fpsyg.2015.01770 (PMC4652008; doi:10.3389/fpsyg.2015.01770)
Supplement: Supplementary file 1 [file DataSheet1.DOCX]

Supplementary Material

Measuring individual differences in decision biases: methodological considerations

**Balazs Aczel*, Bence Bago, Aba Szollosi, Andrei Foldes, Bence Lukacs**

*** Correspondence:** Balazs Aczel: aczel.balazs@ppk.elte.hu

# Study 1

## Extended methods

The tasks of the two decision bias batteries have been adapted from the HB literature after modifying the cover stories and the option structure to satisfy our criteria of sensitivity, construct validity and motivation as described below. Biases listed here and tasks described in **Supplementary Table 1** are ordered the same way as they appeared in the questionnaire.

*Framing effect* refers to the tendency of people to judge objective information differently when it is worded differently. We adapted the classic win/lose framing task from Tversky & Kahneman (1981). We asked the participants in two items to indicate on a 4-point scale their willingness to hire a consulting firm in a described situation. In the first test, one item described that the firm wins applications in 15 out of 20 cases; the other item stated that it loses 5 out of 20 cases. In the second test, if the participant decides to go to court over his/her described financial dispute, then the chance to win the law suit was 33% in the first item, and 66% of losing in the second item. The items were separated by several different questions. For the correct answer, the participants had to indicate the same degree of willingness on the two scales.

*Anchoring Bias* is the tendency to adjust our judgments towards the first piece of information (Tversky & Kahneman, 1974). For the within-subject measure we tested the participants on two items widely separated within the questionnaire. For Test 1, both items required them to choose from a list a range of values which they thought to be the best estimation for the population for Belgium. In the first item they had to answer this question after estimating the population of countries bigger than Belgium; in the second item they had to do it after estimating the population of countries smaller than Belgium. The survey was designed in a way that for the second question, the highest range of the four possible options was the one the participant selected in the first question, and thus was the only correct choice. In Test 2, the test was similarly constructed where the participants had to estimate the price of more and less expensive grocery items, table sugar being in both lists.

*Relativity Problem* is a task first described in Problem 10 of Tversky and Kahneman (1981) where people tend to be less willing to drive 20 minutes to save $5 on a calculator when the original prize was $125 compared to when it was $15. In our Test 1, the participant has to decide for how much reduction would he or she drive 20 minutes when purchasing flight tickets. One of the two separated items in the survey described the price of the ticket, the other one the insurance fees. The price of the ticket was much higher than the insurance fee, although the amounts to be saved were equal for the two items. The choices of the participants were scored as correct if they responded consistently in the two items. In Test 2, we asked the participants to report their willingness to buy a coat for a certain price once after a smaller price increase and later after a larger price increase. We scored their answer biased if they were less willing to buy the same coat if its price was increased from a lower level than if from a higher level.

*Gambler’s fallacy* refers to people’s tendency to believe that if something happens with higher frequency than normal, then on subsequent occasions it should happen less frequently (Tversky & Kahneman, 1971). For both of our versions, we adapted the typical slot machine tasks to describe managerial decision contexts.

*Probability Match* is a probabilistic choice task where instead of the utility-maximizing response, people tend to choose the sub-optimal probability matching strategy (West & Stanovich, 2003). For example, if the probability is 0.7 for option “A” and 0.3 for option “B” then in a series of choices the participants allocate their responses to the two options by matching these probabilities (with success rate of 0.3*0.3+0.7*0.7 = 0.58), instead of maximizing their utility by always choosing the more probable option (with an expected success rate of 0.7). For this task, we adapted the question of Gal and Baron (1996) where they asked the participants what strategy they would use to maximize their success when guessing the color of a die with four red faces and two green faces being rolled 60 times. In our version, the participants play a stock trader with 10 different stocks and it is known that there is 80 % that the price of the stocks will increase during the next month. We know that the price of 8 of the 10 stocks already went up and we asked them about their estimation for the remaining 2 stocks. Only expectation for both of the stocks to increase in price was taken as correct answer. The task in Test 2 followed the same logic.

*Outcome Bias* refers to the error of evaluating a decision based on its outcome. For this task, we reframed the question devised by Baron and Hershey (1988) where a physician's decision to operate on a patient had to be evaluated once when the operation was a success, and once when it was unsuccessful. In our version the management of a company decided to make a big investment. In the case of success it will have a high rate return, but there is a 10% chance that the project fails and the company goes to bankrupt. In one description the outcome was positive, in the other one it was negative. The participants had to evaluate the quality of the decision on a 4-point scale. The choices of the participants were scored as correct if they responded consistently on the two items. To reduce the effect that the participants may remember the first item when responding to the second one, we used different stories for the two cases (e.g., for Test 1 we used biotechnological investment for the first and space tourism for the second question), while keeping the degree of risk equal. Also, the two questions were separated as widely as possible. In Test 2, the first story described an investment opportunity, the second one a tender application with the same forecast, but different outcome.

*Covariation Detection* is a judgment task where the participants receive covariation information and then have to decide whether one component has a positive effect on the other one. For example in the study of Stanovich and West (1998) the participant had to decide whether a drug helps psoriasis for rats based on a 2 × 2 contingency table representing the number of rats that improved and the number that did not improve, given the presence or absence of the drug. In our adaptation in Test 1, the participant is the head of a company that aims to estimate the effectiveness of their new energy bar with data from runners who did or did not consume the bar and whose performance did or did not improve. They had to decide from which of the four cells they needed information to make their assessment. The correct choice would be to select all of the four cells. The task in Test 2 followed the same logic where disregarding any of the four cells could have led them to an unsupported conclusion.

*Sunk cost fallacy* is a maladaptive behavior referring to the tendency of people to continue fruitless endeavor if they have already invested money, time or effort in it (Kahneman & Tversky, 1979). We reframed the task from Experiment 1 in Arkes & Blumer (1985) where the participants had to assume that they bought two weekend ski trip tickets, one for $50 and another one for $100. They knew that they would enjoy the cheaper one more. After buying the tickets, they realized that the two tickets were for the same weekend. They had to choose which one to go on. In Test 1 version, the participant, as the CEO of the company is to send three colleagues to business trainings. The CEO booked two training courses, one more expensive than the other. However, the CEO realizes that the cheaper course will probably be more useful to the staff. After realizing that the two courses are on the same date, the manager has to decide where to send the staff (assuming that they cannot pass on what they have learnt to their colleagues after the course). In Test 2, the participant’s company has two branches. One branch already spent a lot of money on the development of a product and it would need a certain amount to finish the project. The other branch would start the development of a more promising product for which the same amount of money and time would be needed to finish the production. The company has no more money for development than the requested amount. The participant has to decide how to spend the money.

*Conjunction Fallacy* refers to the observation that people tend to violate the normative rule that the probability of a conjunction cannot be greater than the probability of the component events. Adapting the original Linda problem from Tversky and Kahneman (1983) in Test 1 the participant had to play a risk analyst for an IT company with a strong background in software development. They had to judge which option carries smaller risk of failure: a) Constructing an irrigation system for a greenhouse, b) Constructing the irrigation system for a greenhouse, and controlling it by computer. For Test 2, the participants had to put four listed events in descending order of estimated probability. Among the options they read the statement ‘The number of cellphone users will decline’ either alone, or in conjunction with other, more likely events. For both versions, the probability of the conjunct events should not be greater than of their components.

*Regression to the Mean* is the statistical phenomenon that after an extreme event the following measurement will be most probably closer to the mean. It also follows that in uncertainty the mean is always the best prediction. For Test 1 task we simplified the example introduced by Bazerman (2005). We presented the participants with the income data of two companies for four consecutive months. Although the incomes differed between the two companies in each month, their overall average was equal. The participants had to estimate the expected income for the next month. Only the mean values were scored as correct. In Test 2 the participants were told that the management of a chain store offers them an advisory position. Their job is to most accurately estimate next year's turnover rate for each store. The stores are identical with regard to both scale and the offered products, differences in the stores' sales figures are mostly caused by random fluctuation. According to reliable trade forecasts, a 10% increase is expected in the year's total turnover rate. The data for the income of the last year and their mean was provided and the participants had to indicate the most reliable estimation for each store’s income. The correct choice was the mean + 10 %.

*Base Rate neglect task* measures whether people give due respect to base rate information compared to specific information when making decisions. For one version of this task, we adapted the classic Volvo vs. Saab question of Fong, Krantz, & Nisbett (1986) but here the participant is responsible for acquiring new laptops for a company. Reliable statistics favor laptop “A”, while personal experience supports laptop “B”. Only one of the four options suggested buying laptop “A” (correct answer), the other three options preferred laptop “B” or a combination of the two options. For the other version of this task, we adapted the “Broadway production” task from the same source, where the choice is between a candidate who has a great reputation for many years, but shows weak performance at the audition, while others with no outstanding background show better performance at the audition. In our version, the situation is an HR selection where the HR manager has to choose between two candidates for a sales position. The alternative version of this task involved a similar scenario in which the company manager has to dismiss one of the two employees. We scored performance as correct only if the participant answered both of the items correctly. The inclusion of the second item was necessary to keep the chance of correct answer to 25%, equal to the other tasks.

*Monty Hall Problem* is a probability puzzle where people robustly insist on the wrong answer, violating normative decision rules. In the original version of the test (Nalebuff, 1987) a prize is hidden behind one of three doors and the contestant can choose one door to open. Before opening the door, from the unselected doors the host opens an empty one and offers the contestant the opportunity to switch his or her choice to the other remaining door. The contestant typically remains with the first door, although always switching would probabilistically be a better strategy (for an explanation see Kluger & Wyatt, 2004). We adapted the structure of the story into a negotiation and a tender-writing context for the two surveys. For both, the participants had to choose from among one correct and three incorrect options.

*Insensitivity to Sample Size* refers to the observation people disregard the fact that small samples do not comply with the laws of big samples. Adapting the classic hospital problem (Tversky & Kahneman, 1974) to managerial situations, our participants were told in Test 2 that they work for a large automobile manufacturing company, responsible for the acquisition of the manufacturing machinery. They have two machines that produce windshield glass. Machine "A" works 5 hours each day, while machine "B" works 10 hours. Both produce the same percentage of substandard glass. Their job was to estimate which of the two machines are more likely to produce greater proportion of substandard glass in the following month. Only Machine “A” was scored as correct answer. The task in Test 1 followed the same logic.

*Cognitive Reflection Test.* Developed by Frederick (2005), this test is made of three questions where the participants typically provide quick, but incorrect answers. To respond to the questions correctly people have to inhibit their first insights and initiate further deliberate thinking. We did not constrain the time of the participants to answer these questions to keep our results comparable to the previous findings where no time limit was used. Correct answers were scored as 1; incorrect answers were scored as 0. We used the mean performance on the three questions as a composite index, following the practice of previous studies (e.g., Toplak, West, & Stanovich, 2011).

**Supplementary Table 1. HB Tasks for Both Tests in Study 1.** Correct answers are indicated by an asterisk if not stated otherwise.

| **Bias** | **Test 1** | **Test 2** |
| --- | --- | --- |
| **Framing effect** | *Task/1*  You would like to apply for funding for the development of a new technology. A consulting firm offers to write the application for you. The firm is one of the more expensive consulting firms. According to the information available to you, this firm loses applications in 5 out of 20 cases. Based on this information, would you accept the company's offer to write an application?  *Task/2*  You are the owner of a rural hotel. To repair and expand the building, you would like to apply for funding. A consulting firm offers to write the application for you. The firm is one of the more expensive consulting firms. According to the information available to you, the firm wins applications in 15 out of 20 cases. Based on this information, would you accept the company's offer to write the application?  *Answer options for both tasks*  Please indicate on a 4-point scale, how willing you would be to hire the consulting firm.  (1 - Definitely not hire; 4 - Definitely hire)  *Correct answer*  Coherent choices on the tasks. | *Task/1*  You would like to sue one of your former clients because he owes 6 million HUF to your company. The company offers to settle the case out of court. If you accept this arrangement, you will be paid 2 million HUF. However, if you decide to go to court, according to the forecast of your lawyers, you have a 33% chance of winning the lawsuit and getting back the total amount of money within one year.  *Task/2*  One of your subcontractors announces that he got into a difficult financial situation. He now owes 6 million HUF to your company. According to his offer, he would pay you immediately, but he definitely will not be able to pay back 4 million HUF out of the 6 million HUF. If you decline this offer and instead decide to wait, you will have a 33% chance that you do not lose any of your assets, but you will have a 67% chance that the subcontractor cannot pay at all should his company go bankrupt. What is your decision?  *Answer options for both tasks*  Please indicate on a 4-point scale, how willing you would be to accept the offer.  (1 - Definitely not hire; 4 - Definitely hire)  *Correct answer*  Coherent choices on the tasks. |
| **Anchoring bias** | *Task/1*  Estimating the population of Belgium among low anchors.  *Task/2*  Estimating the population of Belgium among high anchors.  *Answer options*  Based on their first choice, different values were presented.  *Correct answer*  Choosing the same value consistently on the tasks. | *Task/1*  Estimating the average price of 1 kg of sugar among low anchors.  *Task/2*  Estimating the average price of 1 kg of sugar among high anchors.  *Answer options*  Based on their first choice, different values were presented.  *Correct answer*  Choosing the same price consistently on the tasks. |
| **Relativity bias** | *Task/1*  You are planning your annual vacation. After you bought the plane tickets you remembered that you also wanted to take out insurance for the trip. At the agency you are informed that the insurance costs 45,000 HUF, but at their nearby office, a 20 minute walk from where you are, you can get the same insurance cheaper.  From the following prices what is the highest that you would walk to the other store for?  *Answer options*  40,000 Ft;  35,000 Ft;  30,000 Ft;  25,000 Ft.  *Task/2*  You are taking a business-trip to New Zealand. You are paying for your own tickets and you decide to buy them from a travel agency. The clerk tells you that the price of the tickets for Business Class is 1,350,000 HUF, but at their nearby office, a 20 minute walk from where you are, you can get the same tickets cheaper.  From the following prices what is the highest that you would walk to the other store for?  *Answer options*  1,345,000 HUF;  1,340,000 HUF;  1,335,000 HUF;  1,330,000 HUF.  *Correct answer*  Consistent choices on both tasks. | *Task/1*  You are shopping for coats. You choose one for 41,000 HUF and you decide that you will buy it the next day. When you arrive to the shop next day you notice that the price increased to 45,000 HUF.  *Task/2*  You are shopping for coats. You choose one for 30,000 HUF and you decide that you will buy it next day. When you arrive to the shop next day you notice that the price increased to 45,000 HUF.  *Answer options for both tasks*  Please indicate on a 4-point scale, how willing you would be to buy the coat.  (1 - Definitely not buy; 4 - Definitely buy)  *Correct answer*  Coherent choices on the tasks. |
| **Gambler’s fallacy** | *Task*  You are responsible for the financial planning of a real-estate agency. Based on the experience of recent years the agency can sell an estate for 60% of their clients. The last 9 clients did not sign a contract.  What do you think is the chance that the 10^th^ client will sign?  *Answer options*  * 60%;  70%;  80%;  90%. | *Task*  You are calculating the budget for a machine that produces components for phones. The machine produces 2% faulty products. Thus far 50 components were produced without faults.  How many do you think will be faulty amongst the next 50 pieces?  *Answer options*  * 1 piece;  2 pieces;  3 pieces;  4 pieces. |
| **Probability match** | *Task*  You are trading with stocks. Based on the analyses the prices of the stocks will increase by 80% over the next month. You have 10 different stocks on the market. After two weeks into the next month you already know that 8 of your stocks have increased.  What do you think will happen to the remaining two?  *Answer options*  Please indicate an answer for each item.   \|  \| Increase \| Decrease \| \| --- \| --- \| --- \| \| Stock #9 \|  \|  \| \| Stock #10 \|  \|  \| | *Task*  You work at a bank and it is your job to suggest investment portfolios to customers that would pay the highest returns. Based on previously collected data, for on average 7 from 10 clients “Portfolio A” is more profitable. However, “Portfolio B” leads to higher profits for 3 from 10 clients.  You can send only one option to each client and because you do not have more information you can only guess as to which bid will be successful for which client. Right now you have 10 interested clients and you have sent “Portfolio A” to 7 of them.  How many of the last 3 clients would you send “Portfolio A” and how many would you send “Portfolio B”?  *Answer options*  “A” for one, “B” for two.  “A” for two, “B” for one.  “B” for all three.  * “A” for all three. |
| **Outcome bias** | *Task/1*  A biotechnology company is considering the development of a new, innovative technology. If the new technology is successfully introduced to the market, the investment will have a high rate of return. However, experts consider the investment very risky, because the company has to take out a large loan to cover development costs. According to the analysts, there is a 10% chance that the project will fail and the whole company will go bankrupt. The management decided to invest in the development, and the project was successful.  *Task/2*  The management of AeroWings Airline is considering starting a space tourism project. If the project is successful, the investment will have a high rate of return, but experts consider the investment very risky, because the financial burden of the project is very serious. According to analysts, there is a 10% chance that the project fails and the whole company goes bankrupt. The company decided to invest in the development, but the project did not turn out to be successful, and the airline went bankrupt because of loans.  *Answer options for both tasks*  Please evaluate the decision of the company on a 4-item scale.  (1 - Definitely not a good decision; 4 - Definitely a good decision)  *Correct answer*  Consistent choices on the 2 tasks. | *Task/1*  One of your acquaintances told you about an interesting investment opportunity. Based on reliable economic analyses, there is a 90% chance that you make an outstandingly high return on this investment. You could only enter into the investment by risking a large amount of money. You decided to enter the investment. The business was successful, and you made a high return.  *Task/2*  You are the owner and the business manager of a small firm. You are invited to a tender. Winning the tender would guarantee sales returns and outstandingly high profit for your firm during the coming years. Applying for the tender, however, requires a lot of money, so you can expect serious losses if the firm loses the tender. Based on experts' forecast, there is a 90% chance that you win the tender. You decided to apply for the tender. You lost the tender, and the company suffered serious losses.  *Answer options for both tasks*  Please evaluate the decision of the company on a 4-item scale.  (1 - Definitely not a good decision; 4 - Definitely a good decision)  *Correct answer*  Consistent choices on the 2 tasks. |
| **Covariation detection** | *Task*  Your company sells dietary supplements for athletes. You would like to test the effectiveness of the new NutroX energy slice before putting it on the market, so you test the product on professional runners. What is the least amount of statistical data you need in order to make certain of the effectiveness of the product?  A) The number of runners that consumed NutroX with their performance improving  B) The number of runners that consumed NutroX without their performance improving  C) The number of runners that did not consume NutroX and their performance improved  D) The number of runners that did not consume NutroX and their performance did not improve  *Answer options*  Only information A;  Information A and B;  Information A, B and C;  * Information A, B, C and D. | *Task*  As the new CEO of a financial advisory company. You notice that if you praise your employees when their performance improves, the following month their performance decreases, and if you call them to account after a decrease in performance then sales figures seem to increase.  How would you modify the benchmarking system solely in light your acquired experience in order to improve overall performance?  *Answer options*  Based on my experience I would place accountability as a central part of leadership for constant performance increase;  * I cannot tell whether praise or calling to account have a greater effect on performance based on my experience;  In case of stagnant and decreasing performance I would keep regular and strict accountability as long as the expected performance increase does not occur;  I continue calling employees to account, but only when the performance decreases. |
| **Sunk cost fallacy** | *Task*  As the CEO of your company, you enroll your employees on finance training course “A”. The total cost of course “A” for the three people is 500.000 HUF. You find out the next week that finance training “B” on the same topic as training “A” would be more useful for your employees. You pay for the total price of training “B”, which is 100.000 HUF. You only realize later on that the two courses are held at exactly the same time. Because both of the courses are experience-based, your colleagues cannot pass on the benefits of the course to other colleagues that did not attend. Which of the courses do you send your colleagues to?  *Answer options*  Send all three colleagues to training "A";  Send two colleagues to training "A", while sending one to "B";  Send one colleague to training "A", while sending two to "B";  * Send all three colleagues to training "B". | *Task*  Your company manufactures audio and lighting equipment. You started the development of a new type of spotlight two years ago. During the two years, the development cost (wages, technology, etc.) has been 70 million HUF, but you need another 30 million HUF for perfecting the product and putting it onto the market, which is expected to happen in a year. If you cannot provide this amount of money, you will have to stop the development, and the project will fail. In the meanwhile, the audio division of your company is also considering development projects. With a 30 million HUF development cost, they would be able to manufacture a new type of loudspeaker within one year. This loudspeaker, according to the forecasts, could be even more profitable on the market than the reflector. The maximum amount of money available for development is 30 million HUF. This means that you can realize only one of the projects. Which project do you decide to finance?  *Answer options*  Spend the whole sum on the development of the spotlight;  * Spend the whole sum on the development of the speaker;  Spend 15 million HUF on each project;  20 million HUF on the development of the speaker, and the rest on the development of the spotlight. |
| **Conjunction fallacy** | *Task*  You are the risk analyst of an IT company. The best developers in the field work at this company. The management decides that the company will expand into new fields. Your task is to decide which of the following projects are the least risky.  Please order the following projects in ascending order according of riskiness.  *Answer options*  Configuring an irrigation system in a greenhouse.  Configuring an irrigation system in a greenhouse and controlling it with a computer.  Configuring an irrigation system in a greenhouse and guttering.  Configuring an irrigation system in a greenhouse and operating it for a year.  *Correct answer*  Every answer combination was scored correct where the first answer was indicated as the least risky. | *Task*  Please order the following events concerning the next two years in ascending order of likelihood.  *Answer options*  The number of cellphone users will decline.  The number of cellphone users will decline and the world market price of gasoline will increase.  The number of cellphone users will decline and the traffic of online shops will increase.  The number of cellphone users will decline and the number of people joining an online social network will increase.  *Correct answer*  Every answer combination was scored correct where the first answer was indicated as the most likely. |
| **Regression to the mean** | *Task*  Your company has two subsidiary companies, “A” and “B”. Although these subsidiary companies are identical with regard to all relevant economic indicators, significantly different incomes were accounted in the past several months. The average income per month is 50,000 dollars for both companies. Please estimate the expected incomes of the next month after reviewing the past incomes.   \|  \| Aug \| Sept \| Oct \| Nov \| \| --- \| --- \| --- \| --- \| --- \| \| A \| 40 M \| 20 M \| 60 M \| 80 M \| \| B \| 20 M \| 80 M \| 60 M \| 40 M \|   *Answer options*  “A”: 40,000, “B”: 20,000;  “A”: 90,000, “B”: 30,000;  * “A”: 50,000, “B”: 50,000;  “A”: 60,000, “B”: 60,000. | *Task*  The management of a chain store offers you an advisory position. Your job is to most accurately estimate next year's turnover rate for each store. The development and marketing budget plan for next year will be based on your estimation. The stores are identical with regard to both scale and the products offered; differences in the stores' sales figures are mostly caused by random fluctuation. According to reliable trade forecasts, a 10% increase is expected in this year's total turnover rate. The stores had the following turnover rates in 2011. Please indicate your best forecasts about the stores' turnover rate in 2012.   \|  \| 2012 \| 2013 \| 2013 \| 2013 \| 2013 \| \| --- \| --- \| --- \| --- \| --- \| --- \| \| Store \|  \| A \| B \| C \| D \| \| 1 \| 22 M \| 24.2 M \| 28.4 M \| 18.4 M \| 24.2 M \| \| 2 \| 24 M \| 26.4 M \| 24.2 M \| 30 M \| 24.2 M \| \| 3 \| 20 M \| 22 M \| 20 M \| 24.2 M \| 24.2 M \| \| Mean \| 22 M \| 24.2 M \| 24.2 M \| 24.2 M \| 24.2 M \|   *Answer options*  A;  B;  C;  *D. |
| **Base-rate neglect** | *Task/1*  As the Chief Financial Officer of a corporation, you are planning to buy new laptops for the workers of the company. Today, you have to choose between two types of laptops that are almost identical with regard to price and the most important capabilities. According to statistics from trusted sources, type “A” is much more reliable than type “B”. One of your acquaintances, however, tells you that the motherboard of the type “A” laptop he bought burnt out within a month and he lost a significant amount of data. As for type “B”, none of your acquaintances have experienced any problems. You do not have time for gathering more information. Which type of laptop will you buy?  *Answer options*  * Order type “A” laptops only;  Order type “B” laptops only;  50% percent of laptops ordered should be type “A”, 50% percent type “B”;  25% percent of laptops ordered should be type “A”, 75% percent type “B”. | *Task/1*  Because the sales returns were lower than expected, you have to cut back on employees at your company. You have decided to dismiss one of the two salesmen. John achieved a stable average performance in the past few years. The other salesman, Tom, had a poorer performance during the past few years. In the past month, however, Tom displayed surprisingly good performance, whereas John's performance dropped back. You have to make a decision today. Who will you dismiss?  *Answer options*  Dismiss John; *Dismiss Tom.  *Task/2*  You are the HR manager of a company and have two candidates for a sales position: Erik and Adam. Judging by their résumés you come to understand that Erik has better sales performance. In the long term he succeeds in 70% of cases. The practice situation yielded the following result in performance: Eric: 40%; Adam: 70%. Who will you promote?  *Answer options*  *Promote Erik; Promote Adam.  *Correct answer*  Coherently choosing by the base rates on the tasks. |
| **Monty Hall problem** | As an investor you want to buy shares in an IT company and you have already started to negotiate with company “A”. The government declared a tender with a generous allowance. For this tender, as well as company “A”, two other companies, “B” and “C”, submitted an application. The decision has already been made, but the winner will be announced later. The tender is expected to guarantee the efficiency of the winning company for the coming years.  You are telling your insider friend that you already have started negotiating with company “A”. Your friends answer is the following:  ‘I cannot tell you which company won, but I can tell you that it is not “B”.’  Which of the following would you choose to win the tender?  *Answer options*  You stay with company “A” as the probabilities are the same.  It does not matter what you do, as you cannot know which company has a greater chance of winning.  * You change to company “C” as the chance of this company winning is higher.  You stay with company “A” as the chance of this company winning is higher. | Your tender-writing company knows that the tenders submitted earlier have a better chance of winning. You are trying to prepare for tenders that have not yet been declared. The government declares new tenders monthly and it leaks that in the next month the tender will follow either of the two plans:  Plan “A”: the tender will concern only medium-sized enterprises for both of the two months.  Plan “B”: the tender will concern medium-sized enterprises in one month and small-sized enterprises in the other month. The order is not known.  In the first month the tender was concerning medium-sized enterprises. What is the probability that it will concern medium-enterprises in the second month?  *Answer options*  50%;  *66,6%;  33,3%;  It cannot be determined. |
| **Insensitivity to sample size** | *Task*  Your task is to predict the expected income of two of the company's divisions. The company's division “A” is twice as big as its division “B”, but they are identical in all other relevant aspects. Which division is more likely to exceed your income prediction by 30%?  *Answer options*  Division “A”;  * Division “B”;  Chances are the same for both divisions;  Cannot provide an answer from the information given. | *Task*  You work for a large automobile manufacturing company and are responsible for the acquisition of the manufacturing machinery. You have two machines that produce windshield glass. Machine “A” works 5 hours each day, while machine “B” works 10 hours. Both produce the same percentage of substandard glass. Your job is to estimate the substandard/ standard glass ratio. Which of the two machines are more likely to produce an unusually high proportion of substandard glass in the following month?  *Answer options*  * Machine “A”;  Machine “B”;  The chance is the same for both machines;  Cannot be determined from the given information. |

**Supplementary Table 2. HB Tasks Sources for each of the Three Tests Batteries in Study 2**

|  | Test 1 | Test 2 | Test 3 |
| --- | --- | --- | --- |
| Framing effect | (Tversky & Kahneman, 1981) | (Bazerman, 1984) | (Druckman & McDermott, 2008) |
| Base-rate neglect | (Tversky & Kahneman, 1974) | (Pennycook, Fugelsang, & Koehler, 2012) | (De Neys & Glumicic, 2008) |
| Covariation detection | (Heijltjes, Van Gog, Leppink, & Paas, 2014) | (De Neys & Van Gelder, 2009) | (Klaczynski, 2001) |
| Insensitivity to sample size | (Toplak, West, & Stanovich, 2011) | (Tversky & Kahneman, 1974) | (Bar-Hillel, 1982) |
| Gambler’s fallacy | (West, Toplak, & Stanovich, 2008) | (Toplak et al., 2011) | (Tversky & Kahneman, 1974) |
| Sunk cost fallacy | (Klaczynski & Cottrell, 2004) | (Arkes & Blumer, 1985) | (Strough, Mehta, McFall, & Schuller, 2008) |

## References

Arkes, H. R., & Blumer, C. (1985). The psychology of sunk cost. *Organizational Behavior and Human Decision Processes*, *35*(1), 124–140.

Bar-Hillel, M. (1982). Studies of Representativeness. In D. Kahneman, P. Slovic, & A. Tversky (Eds.), *Judgment Under Uncertainty: Heuristics and Biases* (pp. 69–83). Cambridge, UK: Cambridge University Press.

Baron, J., & Hershey, J. C. (1988). Outcome bias in decision evaluation. *Journal of Personality and Social Psychology*, *54*(4), 569-579.

Bazerman, M. (2005). *Judgment in managerial decision making*. Hoboken, NJ: John Wiley & Sons.

Bazerman, M. (1984). The relevance of Kahneman and Tversky’s concept of framing to organizational behavior. *Journal of Management*, *10*(3), 333–343.

De Neys, W., & Glumicic, T. (2008). Conflict monitoring in dual process theories of thinking. *Cognition*, *106*(3), 1248–1299.

De Neys, W., & Van Gelder, E. (2009). Logic and belief across the lifespan: the rise and fall of belief inhibition during syllogistic reasoning. *Developmental Science*, *12*(1), 123–130.

Druckman, J. N., & McDermott, R. (2008). Emotion and the framing of risky choice. *Political Behavior*, *30*(3), 297–321.

Fong, G. T., Krantz, D. H., & Nisbett, R. E. (1986). The effects of statistical training on thinking about everyday problems. *Cognitive Psychology*, *18*(3), 253–292.

Frederick, S. (2005). Cognitive reflection and decision making. *The Journal of Economic Perspectives*, *19*(4), 25–42.

Gal, I. (1996). Understanding repeated simple choices. *Thinking & Reasoning*, *2*(1), 81–98.

Heijltjes, A., Van Gog, T., Leppink, J., & Paas, F. (2014). Improving critical thinking: Effects of dispositions and instructions on economics students’ reasoning skills. *Learning and Instruction*, *29*, 31–42.

Kahneman, D., & Tversky, A. (1979). Prospect theory: An analysis of decision under risk. *Econometrica*, *47*(2), 263–292.

Klaczynski, P. A. (2001). Analytic and Heuristic Processing Influences on Adolescent Reasoning and Decision-Making. *Child Development*, *72*(3), 844–861.

Klaczynski, P. A., & Cottrell, J. M. (2004). A dual-process approach to cognitive development: The case of children’s understanding of sunk cost decisions. *Thinking & Reasoning, 10*(2), 147-174.

Kluger, B. D., & Wyatt, S. B. (2004). Are judgment errors reflected in market prices and allocations? Experimental evidence based on the Monty Hall problem. *The Journal of Finance*, *59*(3), 969–998.

Nalebuff, B. (1987). Puzzles: Choose a curtain, duel-ity, two point conversions, and more. *The Journal of Economic Perspectives*, *1*(2), 157–163.

Pennycook, G., Fugelsang, J. A., & Koehler, D. J. (2012). Are we good at detecting conflict during reasoning? *Cognition*, *124*(1), 101–106.

Stanovich, K. E., & West, R. F. (1998). Individual differences in rational thought. *Journal of Experimental Psychology: General*, *127*(2), 161–188.

Strough, J., Mehta, C. M., McFall, J. P., & Schuller, K. L. (2008). Are Older Adults Less Subject to the Sunk-Cost Fallacy than Younger Adults? *Psychological Science*, *19*(7), 650–652.

Toplak, M. E., West, R. F., & Stanovich, K. E. (2011). The Cognitive Reflection Test as a predictor of performance on heuristics-and-biases tasks. *Memory & Cognition*, *39*(7), 1275–1289.

Tversky, A., & Kahneman, D. (1971). Belief in the law of small numbers. *Psychological Bulletin*, *76*(2), 105-110.

Tversky, A., & Kahneman, D. (1974). Judgment under uncertainty: Heuristics and biases. *Science*, *185*(4157), 1124–1131.

Tversky, A., & Kahneman, D. (1981). The framing of decisions and the psychology of choice. *Science*, *211*(4481), 453–458.

Tversky, A., & Kahneman, D. (1983). Extensional versus intuitive reasoning: The conjunction fallacy in probability judgment. *Psychological Review*, *90*(4), 293-315.

West, R. F., & Stanovich, K. E. (2003). Is probability matching smart? Associations between probabilistic choices and cognitive ability. *Memory & Cognition*, *31*(2), 243–251.

West, R. F., Toplak, M. E., & Stanovich, K. E. (2008). Heuristics and biases as measures of critical thinking: Associations with cognitive ability and thinking dispositions. *Journal of Educational Psychology*, *100*(4), 930–941.
