# Supplementary material for: Measuring Individual Differences in Decision Biases: Methodological Considerations
Source: Front Psychol. 2015 Nov 19;6:1770. doi: 10.3389/fpsyg.2015.01770 (PMC4652008; doi:10.3389/fpsyg.2015.01770)
Supplement: Supplementary file 3 [file DataSheet3.DOCX]

***Supplementary Material/2***

**Measuring individual differences in decision biases: methodological considerations**

**Balazs Aczel*, Bence Bago, Aba Szollosi, Andrei Foldes, Bence Lukacs**

*** Correspondence:** Balazs Aczel: aczel.balazs@ppk.elte.hu

**Supplementary Table 3. Polychoric correlation coefficients between the items in Study 1, Test 1**

|  | **Probability match** | **Covariation detection** | **Sunk cost fallacy** | **Conjunction fallacy** | **Regression to the mean** | **Base-rate neglect** | **Monty Hall problem** | **Insensitivity to sample size** | **Framing effect** | **Relativity bias** | **Outcome bias** | **Anchoring effect** |
| --- | --- | --- | --- | --- | --- | --- | --- | --- | --- | --- | --- | --- |
| Gambler’s fallacy | 0,304 | 0,088 | 0,255 | 0,078 | 0,150 | 0,155 | 0,072 | 0,086 | 0,060 | 0,242 | 0,270 | -0,073 |
| Probability match | - | -0,059 | 0,073 | 0,086 | -0,044 | 0,106 | 0,156 | 0,077 | 0,096 | 0,122 | 0,139 | 0,040 |
| Covariation detection | | - | -0,035 | -0,052 | -0,110 | -0,012 | 0,110 | -0,051 | -0,125 | 0,012 | 0,087 | 0,056 |
| Sunk cost fallacy | |  | - | 0,003 | 0,049 | 0,267 | 0,227 | 0,007 | 0,137 | 0,208 | 0,223 | -0,059 |
| Conjunction fallacy | |  |  | - | -0,008 | -0,037 | -0,041 | -0,026 | 0,005 | -0,036 | -0,085 | 0,053 |
| Regression to the mean | |  |  |  | - | 0,028 | 0,035 | -0,038 | -0,002 | 0,138 | 0,054 | 0,047 |
| Base-rate neglect | |  |  |  |  | - | 0,062 | 0,256 | 0,145 | 0,123 | 0,181 | 0,260 |
| Monty Hall problem |  |  |  |  |  |  | - | -0,048 | 0,227 | 0,005 | 0,191 | 0,040 |
| Insensitivity to sample size | |  |  |  |  |  |  | - | 0,113 | -0,009 | 0,006 | -0,126 |
| Framing effect |  |  |  |  |  |  |  |  | - | 0,063 | 0,112 | -0,023 |
| Relativity bias | |  |  |  |  |  |  |  |  | - | 0,247 | 0,074 |
| Outcome bias |  |  |  |  |  |  |  |  |  |  | - | 0,003 |
| Anchoring effect | |  |  |  |  |  |  |  |  |  |  | - |

**Supplementary Table 4. Polychoric correlation coefficients between items in Study 1, Test 2**

|  | **Probability match** | **Covariation detection** | **Sunk cost fallacy** | **Conjunction fallacy** | **Regression to the mean** | **Base-rate neglect** | **Monty Hall problem** | **Insensitivity to sample size** | **Framing effect** | **Relativity bias** | **Outcome bias** | **Anchoring effect** |
| --- | --- | --- | --- | --- | --- | --- | --- | --- | --- | --- | --- | --- |
| Gambler’s fallacy | 0,001 | -0,155 | 0,191 | -0,261 | -0,091 | 0,123 | 0,157 | -0,082 | 0,026 | -0,100 | 0,058 | 0,006 |
| Probability match | - | -0,083 | 0,187 | 0,087 | -0,038 | -0,038 | 0,069 | 0,034 | 0,094 | -0,028 | 0,215 | -0,073 |
| Covariation detection | | - | 0,033 | 0,218 | 0,047 | 0,078 | 0,255 | 0,148 | -0,021 | -0,051 | 0,095 | -0,131 |
| Sunk cost fallacy | |  | - | -0,021 | -0,063 | 0,132 | -0,087 | 0,051 | 0,198 | 0,053 | 0,184 | 0,066 |
| Conjunction fallacy | |  |  | - | 0,067 | 0,215 | 0,066 | 0,029 | -0,158 | -0,078 | 0,117 | 0,077 |
| Regression to the mean | |  |  |  | - | -0,046 | 0,004 | 0,051 | 0,102 | -0,061 | 0,188 | 0,065 |
| Base-rate neglect | |  |  |  |  | - | -0,020 | 0,038 | -0,089 | 0,167 | 0,002 | 0,183 |
| Monty Hall problem |  |  |  |  |  |  | - | -0,128 | -0,122 | 0,197 | 0,062 | -0,101 |
| Insensitivity to sample size | |  |  |  |  |  |  | - | 0,088 | 0,000 | 0,098 | -0,038 |
| Framing effect |  |  |  |  |  |  |  |  | - | -0,052 | 0,167 | -0,026 |
| Relativity bias | |  |  |  |  |  |  |  |  | - | 0,077 | 0,136 |
| Outcome bias |  |  |  |  |  |  |  |  |  |  | - | 0,007 |
| Anchoring effect | |  |  |  |  |  |  |  |  |  |  | - |

**Supplementary Table 5. Polychoric correlation coefficients between the items in Study 2, Test 1**

|  |  | **Base-rate neglect** | **Conjunction fallacy** | **Gambler’s fallacy** | **Covariation detection** | **Sunk cost fallacy** | **Insensitivity to sample size** |
| --- | --- | --- | --- | --- | --- | --- | --- |
| Framing effect |  | 0,022 | 0,005 | 0,242 | 0,089 | 0,091 | -0,003 |
| Base-rate neglect | | - | -0,119 | 0,492 | 0,235 | 0,182 | -0,189 |
| Conjunction fallacy | |  | - | -0,096 | -0,335 | 0,052 | -0,036 |
| Gambler’s fallacy | |  |  | - | 0,245 | 0,281 | -0,406 |
| Covariation detection | |  |  |  | - | 0,038 | 0,012 |
| Sunk cost fallacy | |  |  |  |  | - | -0,100 |
| Insensitivity to  sample size | |  |  |  |  |  | - |

**Supplementary Table 6. Polychoric correlation coefficients between the items in Study 2, Test 2**

|  |  | **Base-rate neglect** | **Conjunction fallacy** | **Gambler’s fallacy** | **Covariation detection** | **Sunk cost fallacy** | **Insensitivity to sample size** |
| --- | --- | --- | --- | --- | --- | --- | --- |
| Framing effect |  | 0,041 | 0,110 | 0,148 | -0,177 | 0,100 | -0,141 |
| Base-rate neglect | | - | 0,181 | 0,136 | 0,181 | 0,282 | -0,160 |
| Conjunction fallacy | |  | - | -0,029 | 0,029 | 0,139 | -0,128 |
| Gambler’s fallacy | |  |  | - | 0,439 | 0,357 | -0,371 |
| Covariation detection | |  |  |  | - | 0,310 | -0,335 |
| Sunk cost fallacy | |  |  |  |  | - | -0,229 |
| Insensitivity to  sample size | |  |  |  |  |  | - |

**Supplementary Table 7. Polychoric correlation coefficients between the items in Study 2, Test 3**

|  |  | **Base-rate neglect** | **Conjunction fallacy** | **Gambler’s fallacy** | **Covariation detection** | **Sunk cost fallacy** | **Insensitivity to sample size** |
| --- | --- | --- | --- | --- | --- | --- | --- |
| Framing effect |  | 0,101 | -0,150 | -0,329 | 0,090 | 0,076 | -0,109 |
| Base-rate neglect | | - | 0,286 | 0,267 | 0,127 | -0,329 | 0,360 |
| Conjunction fallacy | |  | - | -0,206 | 0,004 | -0,168 | -0,365 |
| Gambler’s fallacy | |  |  | - | 0,133 | -0,012 | 0,435 |
| Covariation detection | |  |  |  | - | -0,128 | -0,110 |
| Sunk cost fallacy | |  |  |  |  | - | 0,100 |
| Insensitivity to  sample size | |  |  |  |  |  | - |

**Supplementary Table 8. Factor correlation in Study 1, Test 1**

|  | **Factor 1** | **Factor 2** | **Factor 3** | **Factor 4** |
| --- | --- | --- | --- | --- |
| Factor 1 | - | 0.08 | 0.32 | 0.1 |
| Factor 2 |  | - | -0.04 | 0.01 |
| Factor 3 |  |  | - | 0.31 |
| Factor 4 |  |  |  | - |

**Supplementary Table 9. Factor correlation in Study 1, Test 2**

|  | **Factor 1** | **Factor 2** | **Factor 3** | **Factor 4** |
| --- | --- | --- | --- | --- |
| Factor 1 | - | 0.05 | 0.05 | 0.09 |
| Factor 2 |  | - | 0.03 | 0.01 |
| Factor 3 |  |  | - | 0.28 |
| Factor 4 |  |  |  | - |

**Supplementary Table 10. Factor correlation in Study 2, Test 1**

|  | **Factor 1** | **Factor 2** | **Factor 3** |
| --- | --- | --- | --- |
| Factor 1 | - | 0.1 | 0.22 |
| Factor 2 |  | - | 0.04 |
| Factor 3 |  |  | - |

**Supplementary Table 11. Factor correlation in Study 2, Test 2**

|  | **Factor 1** | **Factor 2** | **Factor 3** |
| --- | --- | --- | --- |
| Factor 1 | - | 0.02 | 0.55 |
| Factor 2 |  | - | 0.1 |
| Factor 3 |  |  | - |

**Supplementary Table 12. Factor correlation in Study 2, Test 3**

|  | **Factor 1** | **Factor 2** | **Factor 3** |
| --- | --- | --- | --- |
| Factor 1 | - | -0.17 | -0.01 |
| Factor 2 |  | - | -0.14 |
| Factor 3 |  |  | - |
